# Healthcare professionals’ perceived barriers and facilitators of implementing clinical practice guidelines for stroke rehabilitation: A systematic review

**DOI:** 10.1177/02692155221141036

**Published:** 2022-12-07

**Authors:** Adrienne Cormican, Shashivadan P Hirani, Eamonn McKeown

**Affiliations:** 1 Occupational Therapy Department, 8948 Kings College Hospital, London, UK; 2 Centre for Health Services Research 4895 City, University of London, London, UK; 3 Health Services Research & Management Division, 152635 School of Health Sciences City, University of London, London, UK

**Keywords:** healthcare professional, clinical practice guideline, stroke rehabilitation, barriers, facilitators

## Abstract

**Objective:**

To identify healthcare professionals’ perceived barriers and facilitators to clinical practice guideline implementation within stroke rehabilitation.

**Data sources:**

CINAHL, MEDLINE, EMBASE, AMED, Cochrane library, Academic Search Complete and Scopus. Additional papers were identified through hand searching.

**Review methods:**

The review followed the Preferred Reporting Item for Systematic Reviews and Meta-Analysis Protocols systematic review approach. Any empirical research that provided qualitative data on healthcare professionals’ perceived factors influencing clinical guideline implementation in stroke rehabilitation was included. One reviewer screened all titles and abstract reviews (*n* = 669). Another two reviewers independently screened 30% of title and abstract reviews, followed by full-text reviews (*n* = 61). Study quality was assessed using the mixed-method appraisal tool.

**Results:**

Data from 10 qualitative, six quantitative and six mixed-method studies published between 2000 and 2022, involving 1576 participants in total, were analysed and synthesised using modified thematic synthesis approach. The majority of participants were therapists *n* = 1297 (occupational therapists, physiotherapists, speech and language therapists). Organisational factors (time constraints, resources) alongside healthcare professionals’ lack of knowledge and skills were the most cited barriers to guideline implementation. Contradictory attitudes and beliefs towards stroke guidelines applicability to real-life clinical practice and their evidence base were reported. Organisational support in the form of training, local protocols, performance monitoring and leadership were reported as perceived facilitators.

**Conclusion:**

Barriers and facilitators are multifactorial and were identified at guideline, individual, team and organisational levels. There is a need to translate perceived barriers and facilitators into implementation interventions especially addressing organisational-level barriers.

## Introduction

Stroke clinical practice guidelines are supported by worldwide evidence of their positive impact on patient outcomes, healthcare costs, quality and effectiveness of stroke services.^1–6^ Such national and international guidelines are crucial components of the healthcare system's quality improvements.^7–17^ They are designed to support decision-making regarding the provision of evidence-based practice and its application^7,9^ thus improving rehabilitation practices. Despite their positive value, they are being underutilised,^18–22^ which can lead to suboptimal patient care with poorer functional recovery.^2,4^ This continues to be a pressing priority in research, management and policy.

An understanding of influential factors has long been advocated as an important component of guideline implementation.^23,24^ Healthcare professionals’ perspectives and experiences represent as such influential factors influencing the effectiveness of clinical guideline implementation.^25^ Comprehensive reviews^26,27^ and meta-reviews^28^ found actual guideline, professional, patient and environmental characteristics all influence generic guideline implementation over a range of healthcare settings.

To date, research consists of studies evaluating stroke guideline adherence,^6^ intervention studies to improve adherence^29–33^ and cross-sectional surveys investigating implementation factors of stroke interventions.^34–37^ Recent systematic reviews on stroke clinical practice guidelines^38,39^ are limited in focus in acute stroke care and neither investigated healthcare professionals’ views about stroke guideline implementation. Halls et al.^40^ systematic review investigates guideline use in stroke rehabilitation. However, it captures only the views of allied health professionals. No study has attempted to systematically analyse published primary studies of all healthcare professionals working in stroke rehabilitation and their perceived barriers and facilitators of guideline implementation to influence the adoption of guidelines in stroke rehabilitation.

Guideline factors were the most weighted domain influencing evidence-based acute stroke therapies^39^ endorsing the need for further focused investigation. Effective stroke rehabilitation is multidisciplinary requiring input from several healthcare professionals. For successful guideline implementation, there is a need to better understand the complexity of changing clinical practice and guideline perceptions of all healthcare professionals working in stroke rehabilitation. Consequently, we conducted a systematic review to investigate and synthesise the current best evidence regarding the barriers and facilitators perceived by healthcare professionals in guideline implementation within stroke rehabilitation.

## Methods

This systematic review was conducted according to the preferred reporting item for systematic reviews and meta-analysis protocols (PRISMA-P) systematic review approach^41^ as outlined in Supplemental Appendix 1 and complies with the Centre of Research and Dissemination Guidelines.^42^ A modified thematic synthesis^43^ was adopted following the guidelines and reporting standards identified by PRISMA.^44^ This approach^43^ has been successfully used within several systematic reviews involving both qualitative and quantitative study designs to address questions about people's perspectives and experiences.^45,46^

**Figure 1.**
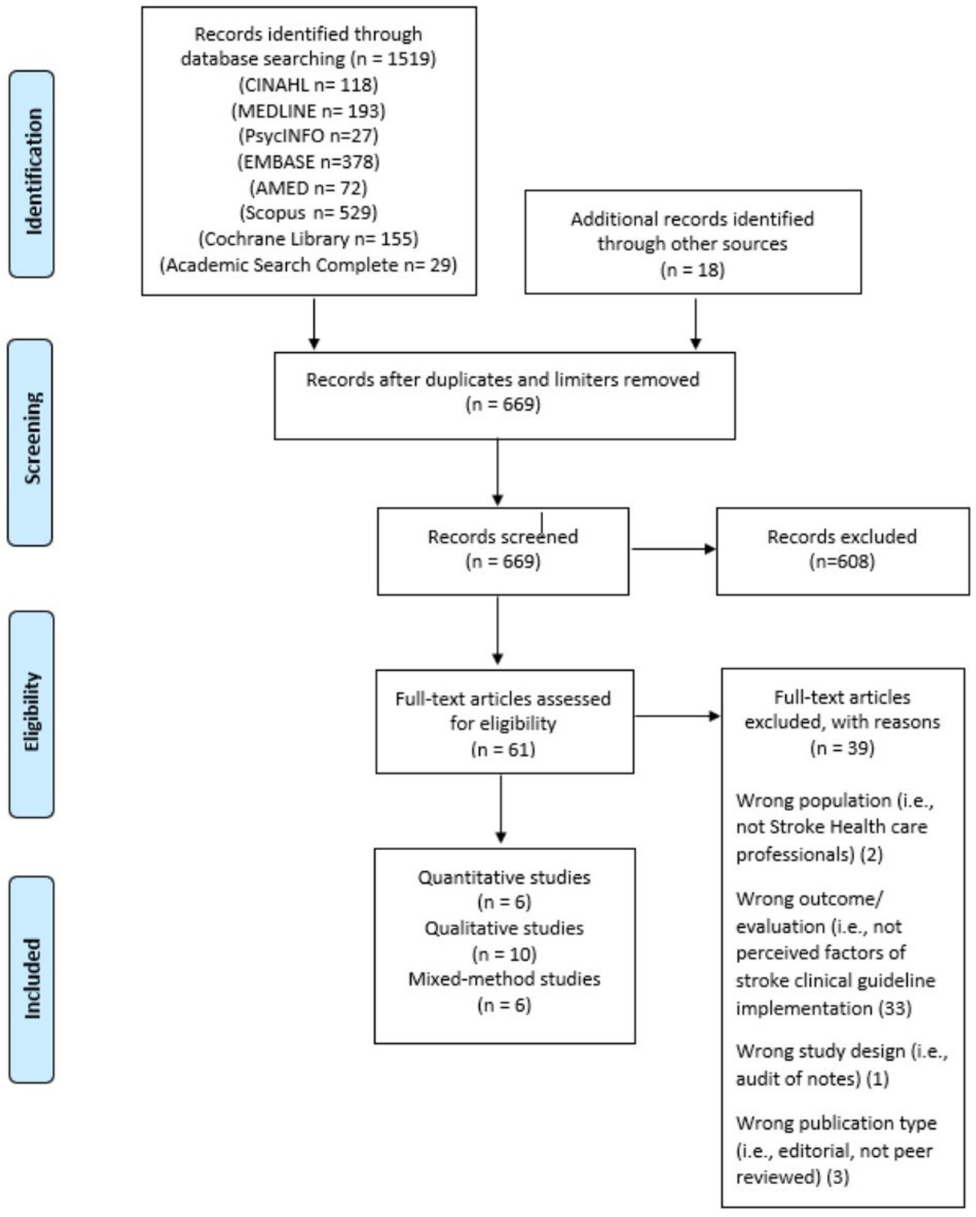
Preferred reporting item for systematic reviews and meta-analysis (PRISMA) flowchart of the study retrieval and section process.

To identify relevant studies, we searched CINAHL, MEDLINE, PsycINFO, EMBASE, AMED, Cochrane Library, Academic Search Complete and Scopus in June 2022. No date limits were applied and databases were searched from the conception date. Additional papers were identified through hand searching reference lists of papers selected for the full-text review. Our search strategy was generated following consultation with an experienced librarian and using search terms developed in previous studies.^39^ A full search strategy is available in Supplemental Appendix 2. This search strategy was adapted for the other literature databases cited in Figure 1. MeSH terms (or equivalent) and free text keywords were used in combination (using Boolean operators) to systematically search the databases mentioned above. Keywords and phrases used included: ‘Clinical practice guideline*’ AND ‘stroke’ OR ‘cerebrovascular accident’ OR ‘stroke rehabilitation’ AND ‘Barrier*’ OR ‘Factor*’ OR ‘Facilitat*’ AND ‘Perception*’ OR ‘Perspective*’.

A study was eligible for inclusion if it met the following selection criteria: (i) a fully published article in a peer-reviewed journal, (ii) qualitative, quantitative (cross-sectional studies) and mixed-methods empirical research, (iii) healthcare professionals working in any stroke rehabilitation setting, and (iv) focus on investigating perceived factors (barriers and/or facilitators) of clinical guideline implementation in stroke rehabilitation. Studies about stroke-specific rehabilitation interventions included in clinical practice guidelines were included if the study described the clinical guideline and its implementation. Studies were excluded if they were: (i) unavailable in English, (ii) a study population primarily of non-healthcare professionals (patients, families, carers), (iii) healthcare professionals’ qualitative perceived factors were not extractable, and (iv) clinical practice guidelines that focused on the medical stroke management or prevention (drug therapy, surgery).

Results were downloaded and imported into Pro-Quest RefWorks citation management system first to remove duplicates and then imported into Rayyan.^47^ Study selection followed a two-step process. Initially, the lead author (AC) screened all titles and abstracts and identified studies for inclusion based on the eligibility criteria checklist. When titles and abstracts had insufficient information to support the screening, a full-text reading was conducted. Two independent reviewers (SH and EM) screened 30% of all titles and abstracts separately, followed by a consensus exercise involving all three authors to resolve discrepancies and arrive at a list of articles for full-text review.^48^ In the second step, the lead author (AC) independently reviewed these full-text selected studies. Studies that met the inclusion criteria were selected for inclusion in the final analysis. The primary reason for the exclusion of full-text selected studies was recorded. See Figure 1.

The following relevant information was extracted from included studies and summated into one document before analysis: author(s), year of publication, country of study, clinical setting and clinical practice guidelines, intervention, study aim, design, data collection methods, participants and key findings on the barriers and facilitators identified to guideline implementation in stroke rehabilitation (see Supplemental Appendix 3). This approach was chosen to provide readers with a broad understanding of each article, how barriers and facilitators were examined, and the type of stroke guideline or implementation intervention being discussed.

As included studies were heterogeneous, the mixed-method appraisal tool^49^ was used to assess the methodological quality of each study retained for this complex systematic review using different evaluation criteria for quantitative, qualitative and mixed-method studies. The specific criteria expose the factors that influence the risk of bias and transparency enabling the lead author (AC) to assess the relevance and rigour of all included studies (see Supplemental Appendix 4). While quality rating was not set as an inclusion criterion, this tool was used to help to inform the confidence with which study results could be interpreted in the synthesis of this review.

The guidelines for thematic synthesis^43^ were followed to identify, extract and synthesise key factors from the selected papers according to our review questions regarding the perceived barriers and facilitators of clinical practice guideline implementation in stroke rehabilitation. All perceived barriers and facilitators found in primary studies were labelled as such in the ‘results’ or ‘findings’ section of study reports. Barriers and facilitators were analysed and discussed collectively. The individual factors that represented barriers and facilitators were represented as initial codes. These initial codes were organised based on recurrency, similarities and differences to construct higher-level ‘descriptive’ themes, which remained close to the primary studies. Finally, overarching analytic themes were developed against the background of the review questions regarding how to promote effective guideline implementation amongst healthcare professionals in stroke rehabilitation. The lead author (AC) was responsible for all data extraction, quality appraisal, and data synthesis. Another author (SH) independently extracted and validated data from five randomly selected studies to ensure consistency with primary studies. SH also blindly assessed for matching themes. The agreement was 100%.

## Results

**Figure 2.**
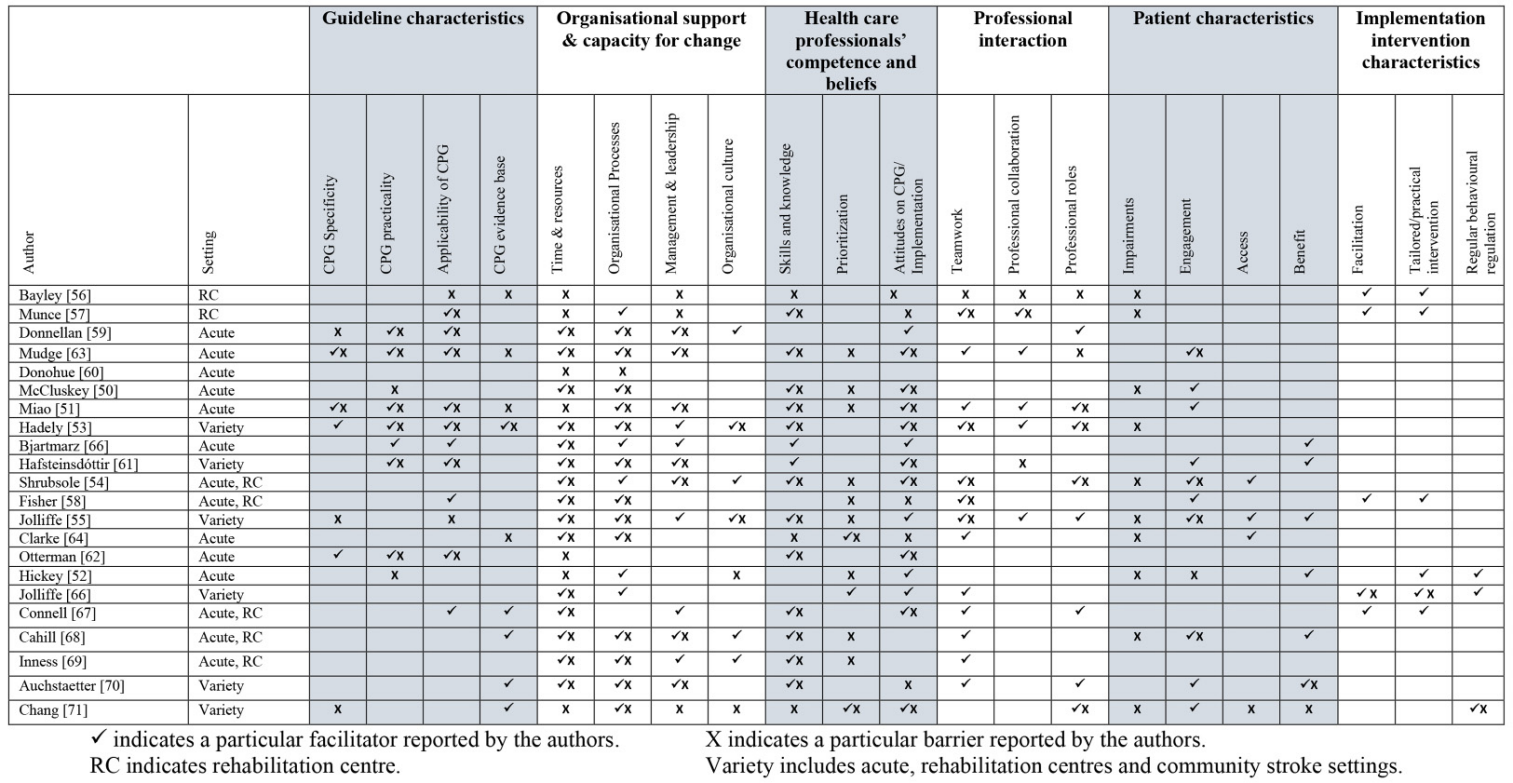
Domain of influential factors (perceived barriers and/or facilitators) under themes.

Twenty-two studies published between 2000 and 2020 met the inclusion criteria.^50–71^ Their key characteristics are presented in Supplemental Appendix 3. Figure 1 depicts a PRISMA flow diagram documenting study selection and exclusion at all stages. The 22 studies provided data from healthcare professionals (*n* = 1576) from seven different countries (Australia, Canada, Ireland, Netherlands, New Zealand, UK, and Iceland). The majority of participants were therapists *n* = 1297 (occupational therapists, physiotherapists, speech and language therapists). The majority of therapists were physiotherapists with over 600 participants, followed closely by speech and language therapists (*n* = 523). Other participants included nurses (*n* = 162), doctors (*n* = 31), managers (*n* = 35), healthcare assistants (*n* = 23), therapy rehab assistants (*n* = 7), social workers (*n* = 2), orthoptics (*n* = 2) and dieticians (*n* = 2). Nine studies targeted single professional disciplines only; physiotherapists,^60,62,70^ speech and language therapists,^51–54,71^ nursing staff^58,61,65^ and other studies involved a combination of therapists.^55,63,66,67,68^ Six studies included a mix of healthcare professionals working as part of multidisciplinary teams.^50,56,57,59,64,69^ Participants recruited in included studies worked in a range of clinical settings along the post-stroke continuum of care – see Figure 2.

Four studies investigated influential factors around national stroke guideline implementation in Ireland^59,60^ and Australia.^51,53^ The remaining studies investigated specific clinical practice guideline recommendation implementation,^50,52,54–58,61–71^ focusing on the upper limb, lower limb, aphasia, balance, communication, swallow, neglect, urinary continence, falls, depression, pain and education. All but one study^52^ examined both perceived barriers and facilitators of the guideline itself and the implementation process/interventions.^56–58,61,64–66^ Barriers and facilitators identified are consistent across quantitative, qualitative and mixed-method studies and were reported sometimes together as a continuum and not exclusively – see Figure 2. Thus, for this present review, healthcare professionals perceived barriers and facilitators to clinical practice guideline implementation were analysed and discussed jointly under themes. Figure 2 displays the six themes and their sub-themes that emerged.

Organisational factors were the most commonly reported factor influencing guideline implementation. Time and resources were the only factors to be reported in all 22 studies, predominantly as a barrier but also as a facilitator if the provision of time was allocated for guideline implementation and adequate availability of resources such as staffing, equipment, assessments, space, funding and educational material. Time constraints link directly with organisational processes and healthcare professionals' prioritisation of guideline recommendations. Unanimously, healthcare professionals' prioritisation of competing clinical and non-clinical tasks negatively interfered with guideline implementation.^50–52,54,55,58,63,64,68,69,71^ This was not just in acute settings where time and the acute medical model is perceived to be a significant limitation but across the continuum of stroke rehabilitation.

A lack of organisational processes acts as a negative influence on guideline implementation^50,51,55,58–61,63,64,68–71^ by way of insufficient training/education, lack of protocols to influence guideline use, lack of specific performance monitoring, poor service structure with daily processes that negatively influence staff prioritisations of recommended interventions and poor documentation systems. Organisational processes perceived as facilitators in 18 studies included organised dissemination of guideline information, consistent training of recommended interventions, alignment of services with guideline recommendations, local protocols, regular performance monitoring and action planning (audits, benchmarking, quality improvement projects), reminders and positive reinforcement for using recommended interventions.^50,51,53–55,57–59,61,63–66,68–71^ Management and leadership were expressed as facilitators by actively supporting the organisational process mentioned above and protecting staff time for implementation creating a high level of organisational priority and commitment to guideline implementation resulting in a positive organisational culture.^53–55,59,67,68,69^

Implementation interventions described in eight studies to increase the uptake of stroke guidelines included change management strategies,^52,56,57^ training and education for that specific stroke rehabilitation guideline,^52,56–58,61,65,66^ auditing,^52,58,65^ facilitators/opinion leaders/champions^57,58,65–67^ and reminders.^57,58,65^ It is not the objective of this systematic review to determine which interventions are most effective, but due to the heterogeneity of the above-mentioned studies, it would be challenging to stipulate which interventions work better over others.

Healthcare professionals' competence (knowledge and skills) was reported as an influential factor in 17 studies. Knowledge and skills barriers identified ranged from a lack of familiarity with and theoretical knowledge behind guideline recommendations to a lack of knowledge and skills of how to modify and transfer guidelines to own local context and implement guideline recommendations.^50,51,53,54,56,57,61–64^ Greater knowledge of the guideline content, clinical skills and experience using recommendations facilitates guideline implementation in practice.^50,51,53–55,62,63,65–67^ Clinical experience and further education were found to be bi-directional. It acted as a facilitator^51,53^ with experienced clinicians being aware of and confident in implementing guidelines but also a barrier^51^ as changing their clinical practice habits can be difficult.

Healthcare professionals' beliefs and attitudes towards guideline implementation were heavily discussed in 19 studies. Eight studies recognised that healthcare professionals’ attitude towards guidelines was bi-directional and a further three studies found guidelines only viewed positively. Clinical practice guidelines were perceived as valuable; as a platform to set practice standards, as an audit tool,^59^ as observed effective interventions,^54,55,65^ for improving patient outcomes,^50^ in ratifying and advocating best practice^51,53,67^ and thus acted as a facilitator for their use. Observing the benefit of using guideline recommendations with patients^52,55,61,64,68,70^ provides healthcare professionals with positive reinforcement and thus acts as a facilitator for continued implementation. Healthcare professionals' negative attitudes related to their own lack of confidence^54,55,67–71^ in providing recommended interventions, patient safety concerns^67,69,70^ and effectiveness of recommended interventions.^50,54,55^ Therapists’ mistrust and wariness regarding the evidence base supporting such guidelines was highlighted as a barrier to implementation in five studies.^51,53,56,63,64^

Contradictory beliefs were identified in relation to guideline characteristics in 13 studies investigating both national and specific guideline recommendations. Guideline applicability was the most commonly described guideline characteristic often negatively perceived^50,51,53,55,57,59,61–63^ as ‘too idealistic’, not feasible, too time intensive for the ‘real world’, linking to a prominent issue of time. Reported facilitators for guideline application into clinical practice included clear, concise, easy to read and navigate recommendations, incorporating prioritised time frames for each recommendation, integrating recommendations into existing routines, and aligning guidelines with working routine practice and models of care. Guideline specificity, practicality and evidence base influenced clinical practice guideline applicability.

Patient characteristics as perceived barriers to guideline implementation were mainly due to the severity of clinical characteristics.^51–57,64,68,71^ The main patient-related facilitator identified was patient and family buy-in and engagement in recommended interventions.^50,51,54,55,58,61,63,68,70,71^ Patient access (their readiness and availability to participate in therapy) was briefly mentioned as a barrier in acute settings^54,64,71^ and as a facilitator across the continuum of stroke settings^55^ with regular access and exposure to appropriate patients.

Professional interactions were the least described barriers and facilitations in included studies. In response to the lack of collaboration as a barrier, many studies described facilitators such as multidisciplinary working parties and guideline committees, and collaboration with patients and with healthcare professionals at different levels.^51,53^ The absence or presence of key authoritative individuals, for example, champions to coordinate implementation efforts were noted as perceived factors.^55,57,58,67,68^ Teamwork was recognised as facilitating guideline implementation through regular team discussions and communication, workload distribution, sharing responsibilities, understanding, engagement and commitment.

## Discussion

The results of this systematic review substantiate the results of earlier stroke^6,39^ and generic literature^23,27,28^ highlighting translation of clinical guidelines into improved clinical practice remains challenging and perceived barriers and facilitators affecting stroke guideline implementation are multifactorial and multi-level. Organisational practice barriers (time, resources and a lack of supporting organisational processes) alongside professional barriers mainly healthcare professionals’ competence (knowledge and skill) in guideline-recommended interventions were the most cited in this review. This is echoed in previous research relating to stroke guidelines and evidence-base practice adherence.^6,39,40^

There remains an over-reliance on identifying and targeting professional level barriers only especially with educational approaches.^63,68^ Education in isolation is not an effective intervention for facilitating practice change.^64,72^ Indeed, this systematic review reflects an emphasis on education as a trialled implementation intervention^52,56–58,61,65^ with a lack of facilitators addressing organisational barriers. One study^66^ builds on the evidence that active and multicomponent approaches are more effective in behaviour change and uptake of clinical guidelines over the educational approach alone.^72^ The findings also draw a parallel to Halls et al.’s^40^ review, which noted that allied health professionals working on stroke are trying to use different active and multifaceted implementation strategies.

Interventions aimed at the organisational and managerial levels can facilitate guideline implementation.^23,73–75^ However, evidence on organisational-orientated interventions remains lacking in comparison with professional-oriented interventions.^76,77^ This review found organisational processes (training, dissemination, local protocols, performance monitoring) were heavily weighted influential factors in guideline implementation. Leadership and management were recognised as facilitators to support organisational processes and create a positive organisational culture. Leadership in the form of opinion leaders/champions/site facilitators are proven effective implementation tools in changing behaviour.^66,73,77,78^ Performance monitoring (auditing and feedback) has shown promise in changing healthcare professionals’ behaviour^31,66,72,79,80^ and found effective in improving stroke clinical guideline application amongst allied healthcare professionals.^40^ Such interventions not only minimise contextual barriers but also promote positive changes in attitudes, behaviours and organisational culture. A greater effort needs to be prioritised to research supporting organisational-level implementation interventions.

Stroke rehabilitation demands collaborative care and thus, team-based implementation interventions should be considered. Two studies in this review^56,57^ found decreased multidisciplinary team communication limited guideline implementation. Multidisciplinary team level interventions can develop a shared understanding, improve team functioning and effect change in guideline implementation.^73^ Systemic challenges in organisational and team level barriers need to be addressed, then individual healthcare professionals will be more empowered to apply guideline recommendations.

The most frequently reported guideline characteristic in this review concerns ‘applicability’ to real-life stroke patients and clinical practice similar to previous literature.^6,23,28,38,39^ This review underlined the prominent issue of healthcare professionals' lack of time, mainly the prioritisation of competing clinical demands and the difficulties associated with impairment severity and co-morbidities. Halls et al.^40^ endorse this finding of a perceived poor fit between stroke rehabilitation guidelines, patient characteristics and healthcare systems' way of working. It is imperative for guideline developers to take into consideration the applicability of guidelines and present practical recommendations. Involving healthcare professionals in guideline development boosts the chances of successful implementation.^81^ An example of important collaborative work in the United Kingdom has been led by the James Lind Alliance.^82^ This national implementation strategy has helped to reveal a shared vision and facilitate collective action towards key priority areas in stroke rehabilitation as identified and shared by stroke clients, carers, researchers and healthcare professionals.

This review found contradictory attitudes and beliefs towards the evidence-base supporting stroke guidelines emphasising a continued relevant factor in guideline implementation research.^6,23,28,38,39^ Jolliffe et al.^83^ and Halls et al.^40^ also report concerns regarding the methodological quality of stroke guidelines evidence base and the implications it poses for their clinical use. Guideline compliance appears to be higher when a strong scientific evidence base underpins them.^84^ Strengthening the evidence base may be influential.

Tailored multicomponent implementation strategies addressing perceived barriers in local contexts remain wanting despite their evidence in eliciting change in clinical practice.^66,72,73,76,85,86^ Only three studies in this review^52,58,66^ investigated potential barriers and facilitators pre-implementation to design their tailored implementation interventions. Implementation interventions informed by behaviour change theories^87^ and stronger study designs are warranted to validate the effectiveness of specific implementation interventions as such evidence remains contradictory due to low-certainty evidence.^72,73^ Additional improvements to implementation design such as design based on known barriers,^85,88–89^ stakeholder engagement,^90^ participatory action research approach^91^ and co-research^92^ should be considered to ensure designs are fit for purpose.

Despite this review following a systematic and transparent approach, it is possible that relevant studies were missed as not every available database was utilised. Databases were selected based on quality and relevancy. The substantial duplication in studies obtained gives a degree of confidence that the main papers that are indexed have been included. Guideline implementation efforts may have occurred as local quality improvement projects and therefore, were not published in peer-reviewed journals. Boundaries set around inclusion criteria for this review were necessary and as a minimum quality standard. Peer-reviewed sources are likely to be larger and more impactful to the broader profession. The heterogeneity of the studies including the thin data and description of perceived barriers and facilitators in quantitative and mixed-method studies may have been a barrier. However, the included studies show a reasonable consistency of results, which was drawn out using an explicit approach to appraise, combine and synthesise complex data and thus adds to the confidence in the conclusions of this review.

The current review is the first to specifically detail healthcare professionals perceived barriers and facilitators of implementing clinical practice guidelines in stroke rehabilitation. The findings add to the knowledge base and can inform future clinical practice guideline implementation interventions in stroke rehabilitation. The results of this review stress the importance of healthcare professionals’ experiences, views and their input in guideline policy and clinical management to address barriers and develop strategies to optimise the implementation of guidelines in stroke rehabilitation. Understanding perceived barriers and facilitators will enable managers, commissioners, and policymakers to deliver more focused and realistic recommendations, support and guidance to healthcare professionals.

**Clinical messages**

- Tailored multifaceted implementation approaches addressing local contextual barriers can improve guideline implementation.
- Perceived organisational facilitators for guideline implementation include consistent training, services and local protocols aligned with guideline recommendations, regular performance monitoring and action planning.
- Effective management and leadership (guideline champions) can facilitate organisational commitment and priority to guideline implementation.

## Supplemental Material

sj-docx-1-cre-10.1177_02692155221141036 - Supplemental material for Healthcare professionals’ perceived barriers and facilitators of implementing clinical practice guidelines for stroke rehabilitation: A systematic review Click here for additional data file. Supplemental material, sj-docx-1-cre-10.1177_02692155221141036 for Healthcare professionals’ perceived barriers and facilitators of implementing clinical practice guidelines for stroke rehabilitation: A systematic review by Adrienne Cormican, Shashivadan P Hirani and Eamonn McKeown in Clinical Rehabilitation
