# Supplementary material for: Healthcare professionals’ perceived barriers and facilitators of implementing clinical practice guidelines for stroke rehabilitation: A systematic review
Source: Clin Rehabil. 2022 Dec 7;37(5):701–12. doi: 10.1177/02692155221141036 (PMC10041573; doi:10.1177/02692155221141036)

**Appendices**

Supplementary appendix 1- PRIMSA-P

Supplementary appendix 2- PRISMA 2009 checklist

Supplementary appendix 3- Full search strategy

Supplementary appendix 4- Data extraction table

Supplementary appendix 5- Mixed method appraisal tool

**Supplementary Appendix 1: PRISMA-P**

**RESEARCH PROPOSAL**

Written in line with PRISMA-P (Preferred Reporting Items for Systematic review and Meta-Analysis Protocols) 2015 statement (Shamseer et al., 2015)

**ADMINISTRATIVE INFORMATION**

**Title:** Health care professionals’ perceived barriers and facilitators of implementing clinical practice guidelines for stroke rehabilitation: A systematic review.

**Registration:** Not registered.

**Authors:** Adrienne Cormican (Corresponding author) Health Services Research & Management Division, School of Health Sciences, City, University of London, 1 Myddelton Street, London, EC1V 0HB. Adrienne.cormican@city.ac.uk

Dr Shashivadan P Hirani (Corresponding author) Centre for Health Services Research, City, University of London, Northampton Square, London, EC1V 0HB. shashi.hirani@city.ac.uk, Telephone: 02070400880

Dr Eamonn McKeown Health Services Research & Management Division, School of Health Sciences, City, University of London, 1 Myddelton Street, London, EC1V 0HB. Eamonn.McKeown.1@city.ac.uk

**Contributions**  AC is the CI leading protocol development, analysis and dissemination. SH and EM are first and second reviewers. All authors will contribute to data interpretation and article drafts.

**Amendments**  Important protocol amendments post registration will be recorded and included in dissemination.

**Support**  This study was funded by the National Institute for Health Research (NIHR) and the Stroke Association as part of the Pre-doctoral Clinical Academic Fellowship (PCAF) scheme. AC is completing a Masters in Clinical Research at City, University of London as part of her PCAF award. Other authors are academic staff at City, University of London.

**INTRODUCTION**

**Rationale**

Globally over the past 3 decades, there has been a considerable increase in stroke-related disability and is expected to continue to grow as a major global societal burden (Feigin et al 2009; Platz, 2019). As a result, efforts to decrease this rising stroke burden has led to significant generation of research and evidence to support stroke preventative and rehabilitative interventions. One such tool to allow translation and facilitation of research-evidence into health care is clinical practice guidelines (CPGs). Over the last 20 years, there has been a surge in countries establishing stroke CPGs to standardize stroke care driven on and supported by the evidence of their positive impact on patient outcomes (survival and recovery), healthcare costs, quality and effectiveness of stroke services, (Duncan et al., 2002; Cadilhac et al., 2008; Brusamento et al., 2012, Hubbard et al., 2012, Stroke Unit Trialists’ collaboration, 2013, Donnellan et al 2013).

CPGs are systematically developed, unbiased statements summarising current published research (Field and Lohr, 1992; Grol and Grimshaw, 2003). They are intended to optimise patient care (Institute of Medicine, 2011) but are also designed to support health care professionals (HCPs) application of evidence-based practice (EBP) in specific clinical setting (Grol and Grimshaw, 2003; Van der Wees et al., 2008, Royal College of Physicians, 2016; Stroke Foundation, 2017) thus promoting the delivery of high quality, cost-effective care. Stroke CPGs were designed to improve rehabilitation practices and assist clinicians in decision making regarding the provision of evidence-based interventions and care (Field and Lohr, 1992; Royal College of Physicians, 2016; Stroke Foundation, 2017).

Despite their positive value, impact and strength of evidence to support them, CPGs are known to have poor and variable compliance rates by HCPs in daily patient care (Cochrane et al., 2007). Specific to stroke, studies by the Harris et al. (2010), National Stroke Audit (2012), Hubbard et al., 2012, Stroke Foundation (2018), Sentinel Stroke National Audit Programme (2019), The Scottish Stroke Care Audit (2015), Korner-Bitkensky, Desrosiers and Rochette (2008) conclude that CPGs are being underutilised. While adherence to Stroke CPGs has increased somewhat, translation of research-evidence into clinical practice through CPGs continues to be a pressing priority for health professionals in policy, management and research owing to the longstanding evident gap between research and stroke clinical practice (Holloway, Benesch and Rush, 2000). This “evidence-practice” gap leads to suboptimal care for many patients. Lack of adherence with CPGs could reflect the way they are implemented or factors affecting implementation.

There is a wealth of literature discussing CPG implementation from a generic perspective. Comprehensive reviews (Prior, Guerin and Grimmer-Somers, 2008) and meta-reviews (Francke et al., 2008) found actual guideline, professional, patient and environmental characteristics all influence guideline implementation over a range of health care settings. Grimshaw, Eccles and Tetroe (2004) review evaluated the effectiveness of strategies from 235 studies and later Francke et al (2008) and Prior, Guerin and Grimmer-Somers (2008) concluded in accordance with his findings that multifaceted tailored interventions including interactive education, clinical reminders and active engagement from clinicians are required for successful CPG implementation. Medves et al (2010) more recently focusing on team-based practice added to the CPG implementation evidence-base.

Regarding the evidence-based practice (EBP) within stroke care, several studies have attempted to shed light on barriers and facilitators of applying EBP in stroke rehabilitation through examining implementation interventions through cohort study design (Kristensen, Nymann and Konradsen, 2016; Bjartmarz, Jónsdóttir and Hafsteinsdóttir, 2017; Ajimsha, Kooven and Al-Mudahka 2019). More focused to stroke CPG implementation, Bayley et al (2012) piloted an implementation study and examined factors that influence allied health professionals stroke CPGs implementation and found similar influential factors as Francke et al (2008) and Prior, Guerin and Grimmer-Somers (2008).

Increasingly, experts in EBP implementation advocate using an understanding of barriers and facilitators, views and attitudes as a basis for planning implementation strategies (Cabana et al., 1999). Evidence from Mickan et al (2010) highlights the important role HCPs behaviours (awareness, agreement, adoption of CPGs) play in influencing CPG implementation. There has been an increase in worldwide research over the past number of years investigating HCPs views and perceptions towards CPG implementation in stroke rehabilitation which can be perceived as determinates for HCPs behaviours and actions concerning CPG implementation. The benefits of this valuable information acquired can be represented as a distinguishing factor influencing the effectiveness of CPG implementation.

To date, no study has attempted to systematically analyze published primary studies on HCPs perceived barriers and facilitators of implementing CPGs in stroke rehabilitation. While, Donnellan et al (2013) looked at studies that evaluated adherence of generic and specific stroke CPGs and intervention studies to improve adherence of CPGs, they concluded that limited number of studies assessed HCP perceived supports and hindrances regards CPG implementation. Only four studies ascertained HCP views through surveys and only in relation to implementing CPGs on specific stroke interventions such as: outcome measure use (Van Peppen et al., 2008); thrombolysis (Van der Weijden et al., 2004); early mobilisation (Arias and Smith, 2007); depression screening (Hart and Morris, 2008) advocating the need for further investigation of HCPs views on generic CPGs in stroke rehabilitation.

The most recent systematic review by Baatiema et al (2017) on HCPs views is limited in focus to four acute stroke care (stroke unit care, thrombolysis administration, aspirin usage, and decompressive surgery) similar to Craig et al (2016) systematic review around triage and treatment of stroke in Emergency departments. Neither systematic review investigated HCPs views about stroke CPG’s implementation. Baatiema et al (2017) highlights that guideline factors were the most weighted domain of barriers and enablers of evidence-based acute stroke therapies endorsing the need for further focused investigation into HCP perceived barriers and facilitators of stroke CPG implementation.

Given the evidence that implementation of stroke CPGs leads to better quality of care and improved stroke outcomes, it is imperative to improve recommended CPGs implementation in stroke rehabilitation. The gap between the guideline recommendations and actual clinical practice in stroke rehabilitation is likely to be a result of a number of factors which together inﬂuence the HCPs choices and actions.

Therefore, the purpose of this systematic review is to report on a synthesis of current best evidence regarding HCPs perceived barriers and facilitators to implementing stroke CPGs within stroke rehabilitation. Such a review will add to the implementation evidence-based literature and is important for informing future quality improvement and knowledge translation research aiming to increase the adherence of stroke CPGs ensuring the highest possible standard of practice in stroke rehabilitation. This information may build on knowledge generated by previous works, stressing the importance of HCPs views and input in policy and management to address these barriers and develop strategies to optimise uptake of CPGs in stroke rehabilitation to improve stroke survivor’s outcomes.

**Objectives**  To synthesise the existing evidence of HCP’ perspectives of CPG implementation in stroke rehabilitation.

(i) What are health care professionals’ perceptions of Stroke clinical practice guidelines?

(ii) What do health care professionals think hinders them from implementing clinical practice guidelines in stroke rehabilitation?

(iii) What do health care professionals think helps them to implement Clinical practice guidelines in Stroke rehabilitation?

(iv) To identify and discuss common and different perceived barriers and facilitators amongst different professions internationally?

**METHODS** Design and methods used for this systematic review were developed according to recommendations from the Preferred Reporting Items for Systematic Reviews and Meta-Analyses (PRISMA) (Moher et al., 2009) and Preferred Reporting Items for Systematic Reviews and Meta-Analyses Protocols (PRISMA-P) statement (Shamseer et al., 2015) and the comply with Centre of Research and Dissemination Guidelines (CRD, 2009). Eligibility criteria were informed using the SPIDER framework (Cooke, Smith and Booth, 2012).

**Eligibility Criteria**

See Table 1 for Eligibility of screened references. (S) Sample: Health care professionals working in stroke rehabilitation (acute, community, long term care) (PI) Phenomenon of Interest: perspectives, views of clinical practice guidelines and their implementation in stroke rehabilitation. (D) Design: Observational study design (cross sectional studies) and qualitative or phenomenological study design, mixed method design. (E) Evaluation: studies identifying or investigating factors, barriers or facilitators, views, attitudes, experiences or perspectives of clinical practice guideline implementation in stroke rehabilitation (R) Research Type: qualitative, quantitative and mixed-methods research could be searched for.

**Information sources** The search will employ sensitive topic-based strategies designed for each database from inception to June 2020. There will be no geographical or year of publication restrictions but a restriction on language - English only studies.

- Electronic Databases: Cumulative Index to Nursing and Allied Health Literature (CINAHL) via EBSCO, MEDLINE, Excerpta Medica database (EMBASE), PsycINFO, the Allied and Complementary Medicine Database (AMED), Cochrane Library, Academic Search Complete (available EBSCOhost), Physiotherapy Evidence Database (PEDro) and Scopus.
- Selected official internet sites and indexes: Turning Research into Practice, National Stroke Association, RCN Clinical Topics: Stroke, Stroke Association, Google Scholar.
- Hand search reference lists and bibliographies of key papers which met the inclusion criteria
- Contact with study authors of key papers identified from the reference lists of included studies.

**Search strategy**

The search strategy was derived from a scoping search and expertise in the subject field. The search strategy will include filters comprised of Medical Subject Heading (MeSH) terms, additional key terms and relevant synonyms for each concept, related to the review question and combined with BOOLEAN operator “OR” and “AND”. Detailed information of the search terms used are presented as an example in Figure 1.

Figure 1.

| **Main search terms** | **Relevant associated words/concepts** |
| --- | --- |
| Clinical Guidelines | “Clinical practice guideline*”  “National clinical guideline*”  “clinical guideline*”  “Evidence-based recommendation*” |
| Stroke  Stroke rehabilitation | “cerebrovascular accident”  “CVA”  “stroke rehabilitation” |
| Implementation | Adhere*  Implement*  Establish*  Appl* |
| Barriers | Barrier*  Challenge*  Difficult*  Prevent*  Obstacle*  Inhibitor*  Limitat*  Factor* |
| Facilitators | Drivers*  Facilitat*  Enabler*  Motivator* |
| View | Perception*  Perspective*  View* |

**Data management** Records will be managed through RefWorks and Rayyan QCRI (Ouzzani et al., 2016), specific software for managing systematic reviews through all stages of the process from bibliographic management, screening, coding and right through to synthesis. Duplicate studies will be recorded and merged to remove duplicates through both RefWorks and Rayyan QCRI.

**Selection process** Initially, one main reviewer (AC) will screen information sources independently and assess identified studies for inclusion based on relevance of study titles and abstracts to the review aim using an eligibility criteria checklist developed from inclusion and exclusion criteria. A full text reading will be conducted when titles and abstracts have insufficient information and cannot be clearly excluded (CRD, 2009) following discussion between all authors. Using a pre-designed eligibility checklist, one reviewer (AC) will independently assess the full text of all obtained studies against the eligibility criteria and an eligibility code will be recorded. Full text studies that do not meet the inclusion criteria will be excluded and primary reason for exclusion will be recorded.

30% of the abstracts and titles will be reviewed separately and independently by two other authors (SH and EM), followed by a consensus exercise to resolve discrepancies and arrive at a list of articles for full text review. A second author (SH) will independently review a randomly selected sample (n=5) of these full-text selected studies to ensure they met eligibility criteria. A third author (EM) will mediate in the event of disagreement between the two reviewers (AC and SH) following discussion (Furlan et al., 2009). Results of full text will also be shared with the remaining reviewer (EM) to validate their eligibility. Full text studies that meet the inclusion criteria will be selected for inclusion in final analysis.

**Data collection process** Using a piloted data extraction form (evidence table), one reviewer (AC) will extract data independently from full text included studies (CRD, 2009). A second reviewer (SH) will independently validate the extracted data of a randomly selected sample of studies (n=5) to ensure consistently with primary studies. SH will also blindly assess for matching themes. Any disagreements will be resolved by a third reviewer (EM) or by achieving consensus through discussion.

**Data items** Using a piloted data extraction form, key data to be extracted will include the following summary:

- Publication details: Author(s), year of publication, title of paper, journal and country in which study was conducted.
- Study design: study type, design type, study aims, intervention, recruitment method and data collection methods.
- Participants: sample size and characteristics
- Clinical setting: unit/setting and clinical practice guideline characteristics
- Results: key findings on perspectives of stroke CPG implementation, description of perceived barriers and facilitators to CPG implementation with reported stakeholders noted, any comparison among participant, clinical settings, and CPGS characteristics.

**Risk of bias in individual studies** Risk of bias and methodological quality for each full text individual study included will be independently assessed by lead author (AC). As the included studies may be heterogeneous, the quality of mixed-method research and risk of bias will be assessed using the Mixed method Appraisal tool (MMAT) (Pluye et al., 2013). This tool uses different evaluation criteria for quantitative, qualitative, and mixed-method studies and will be piloted prior to use.

**Data synthesis**

It is unlikely that a meta-analysis will be possible based on findings of scoping search. Thematic Synthesis (Thomas and Harden, 2008) will be applied to identify, extract, synthesise and develop key descriptive themes, which remain close to the primary studies findings. Findings from both quantitative and qualitative data will be summarised in a descriptive account that addresses the review questions to provide a better understanding of HCPs perceived barriers and facilitators around stroke CPGs in stroke rehabilitation. Barriers and facilitators will be analysed and discussed collectively.

**Results**

Results of the search and study selection process will be presented in a PRISMA flow chart (Moher et al., 2009). Relevant data extracted from eligible studies will be presented as an evidence table. Where appropriate data will be presented in tables and graphics. A detailed discussion of the limitations of the included studies and their implications on the findings will be provided. We will identify gaps in knowledge and provide suggestions for future research.

**Dissemination**  Findings from this systematic review will form part of AC Master’s dissertation. The review will be prepared following PRISMA reporting standards for submission to a peer-reviewed journal with focus on clinical practice in stroke rehabilitation and presented at conferences.

**References**

Shamseer L, Moher D, Clarke M, et al. Preferred reporting items for systematic review and meta-analysis protocols (PRISMA-P) 2015: elaboration and explanation. *BMJ* 2015; 349: g7647.

Feigin VL, Lawes CMM, Bennett DA, et al. Worldwide stroke incidence and early case fatality reported in 56 population-based studies: a systematic review. *Lancet Neurol* 2009; 8: 355–69.

Platz T. Evidence-based guidelines and clinical pathways in stroke rehabilitation—an international perspective. *Front Neurol* 2019; 10: 200.

Duncan PW, Horner RD, Reker DM, et al. Adherence to post-acute rehabilitation guidelines is associated with functional recovery in stroke. *Stroke* 2002; 33: 167-178.

Cadilhac DA, Pearce DC, Levi CR, et al. Improvements in the quality of care and health outcomes with new stroke care units following implementation of a clinician-led, health system redesign programme in New South Wales, Australia. *Qual Saf Health Care* 2008; 17: 329-33.

Brusamento S, Legido-Quigley H, Panteli D, et al. Assessing the effectiveness of strategies to implement clinical guidelines for the management of chronic diseases at primary care level in EU Member States: a systematic review. *Health Policy* 2012; 107: 168–183.

Hubbard IJ, Harris D, Kilkenny MF, et al. Adherence to clinical guidelines improves patient outcomes in Australian audit of stroke rehabilitation practice. *Arch Phys Med Rehabil* 2012; 93: 965–971.

Stroke Unit Trialists' Collaboration. Organised inpatient (stroke unit) care for stroke. *Cochrane Database of Syst Rev* 2013; 9: CD000197.

Donnellan C, Sweetman S and Shelley E. Health professionals’ adherence to stroke clinical guidelines: a review of the literature. *Health Policy* 2013; 111: 245–263.

Field MJ and Lohr K. *Guidelines for Clinical Practice: from development to use.* Washington DC: National Academic Press, 1992.

Grol R and Grimshaw J. From best evidence to best practice: Effective implementation of change in patients’ care. *Lancet* 2003, 362, 1225–1230.

Institute of Medicine (US) Committee on Standards for Developing Trustworthy Clinical Practice Guidelines. *Clinical practice guidelines we can trust*. Washington, DC: The National Academics Press, 2011.

Van der Wees PJ, Jamtvedt G, Rebbeck T, et al. Multifaceted strategies may increase implementation of physical therapy clinical guidelines: a systematic review. *Aust J Phys Ther* 2008; 54: 233–241.

Royal College of Physicians, Intercollegiate Stroke Working Party. National clinical guideline for stroke. The Fifth edition. London, UK, 2016.

Stroke Foundation. Clinical Guidelines for Stroke Management 2017. Stroke Foundation, Melbourne, Australia, 2017.

Cochrane LJ, Olson CA, Murray S, et al. Gaps between knowing and doing: understanding and assessing the barriers to optimal health care*. J Contin Educ Health Prof* 2007; 27: 94–102.

Harris D, Cadilhac DA, Hankey GJ, et al. National stroke audit: the Australian experience. Clinical Audit. 2010; 2: 25–31.

National Stroke Foundation. National Action Plan for Stroke. Melbourne, Australia, Melbourne, Australia, Dec 2012.

Stroke Foundation. National Stroke Audit - Rehabilitation Services Report, Melbourne, Australia, Oct 2018.

Sentinel Stroke National Audit Programme (SSNAP). Acute Organisational Audit Report 2019 King’s College London, UK, Dec 2019.

The Scottish Stroke Care Audit (SSCA) 2015 Annual National Report Stroke services in Scottish Hospitals, Edinburgh, Scotland, 2015.

Korner-Bitkensky N, Desrosiers J and Rochette A. A national survey of OT practices related to participation post-stroke. *J Rehabil Med* 2008; 40(4): 291 – 7.

Holloway RG, Benesch C and Rush SR. Stroke prevention: narrowing the evidence-practice gap. *Neurology* 2000; 54(10): 1899-906.

Prior M, Guerin M and Grimmer-Somers K. The effectiveness of clinical guideline implementation strategies – a synthesis of systematic review findings. J *Eval in Clin Pract* 2008; 14: 888–97.7

Francke A, Smit M, de Veer A, et al. Factors influencing the implementation of clinical guidelines for health care professionals: a systematic meta-review. BMC Med Inform Decis Mak 2008; 8 :38.

Grimshaw J, Eccles M and Tetroe J. Implementing clinical guidelines: current evidence and future implications. *J Contin Educ Health Prof* 2004; 24(1): S31-7.

Medves J, Godfrey C, Turner C, et al. Systematic review of practice guideline dissemination and implementation strategies for healthcare teams and team-based practice. *Int J Evid Based Healthc* 2010; 8: 79–89.

Kristensen N, Nymann C and Konradsen H. Implementing research results in clinical practice- the experiences of healthcare professionals. *BMC Health Service Research* 2016; 16: 48.

Bjartmarz I, Jónsdóttir H and Hafsteinsdóttir TB. Implementation and feasibility of the stroke nursing guideline in the care of patients with stroke: a mixed methods study. BMC Nursing 2017; 16: 72.

Ajimsha MS, Kooven S and Al-Mudahka N. Adherence of physical therapy with clinical practice guidelines for the rehabilitation of stroke in an active inpatient setting, *Disabil Rehabil* 2019; 41: 15.

Bayley MT, Hurdowar A, Richards CL, et al. Barriers to implementation of stroke rehabilitation evidence: findings from a multi-site pilot project. *Disabil Rehabil* 2012, 34(19): 1633-1638.

Cabana MD, Rand SC, Powe NR, et al. Why don’t physicians follow clinical practice guidelines? A framework for improvement. *JAMA* 1999; 282(15): 1458-1465.

Van Peppen RPS, Maissan FJS, Van Genderen FR, et al. Outcome measures in physiotherapy management of patients with stroke: a survey into self-reported use, and barriers to and facilitators for use*. Physiother Res Int* 2008; 13(4): 255–270.

Van der Weijden T, Hooi JD, Groi R, et al. A multidisciplinary guideline for the acute phase of stroke: barriers perceived by Dutch neurologists. *J Eval Clin Pract* 2004; 10(2); 241–246.

Arias M and Smith LN. Early mobilization of acute stroke patients. *J Clin Nurs* 2007; 16: 282–8.

Hart S and Morris R. Screening for depression after stroke: an exploration of professionals’ compliance with guidelines. *Clin Rehabil* 2008; 22: 60–70.

Baatiema L, Otim ME, Mnatzaganian G, et al. Health professionals’ views on the barriers and enablers to evidence-based practice for acute stroke care: a systematic review. *Implement Sci* 2017; 12: 74.

Craig LE, McInnes E, Taylor N, et al. Identifying the barriers and enablers for a triage, treatment, and transfer clinical intervention to manage acute stroke patients in the emergency department: a systematic review using the theoretical domains framework (TDF). *Implement Sci* 2016; 11: 57.

Moher D, Liberati A, Tetzlaff J, et al. Preferred reporting items for systematic reviews and meta-Analyses: The PRISMA Statement. *PLoS Med* 2009; 6(7): e1000097.

Centre for Reviews and Dissemination (CRD) Systematic Reviews, CRD’s guidance for undertaking systematic reviews in health care. CRD, University of York, UK, Jan 2009.

Cooke A, Smith S and Booth B. Beyond PICO: The SPIDER Tool for Qualitative Evidence Synthesis. *Qual Health Res* 2012; 22(10): 1435-1443.

Ouzzani M, Hammady H, Fedorowiczet Z, et al. Rayyan—a web and mobile app for systematic reviews. *Syst Rev* 2016; 5: 210.

Furlan AD, Pennick V, Bombardieret C, et al. Updated Method Guidelines for Systematic Reviews in the Cochrane Back Review Group. *Spine* 2009; 34: 1929 –1941.

Pluye P. Critical appraisal tools for assessing the methodological quality of qualitative, quantitative and mixed methods studies included in systematic mixed studies reviews [Letter]. *J Eval Clin Pract* 2013; 19(4): 122.

Thomas J and Harden A. Methods for the thematic synthesis of qualitative research insystematic reviews. *BMC Med Res Methodol* 2008; 8: 45.

Moher D, Liberati A, Tetzlaff J, et al. Preferred reporting items for systematic reviews and meta-Analyses: The PRISMA Statement. *PLoS Med* 2009; 6(7): e1000097.

**Supplementary Appendix 2: PRISMA 2009 Checklist**


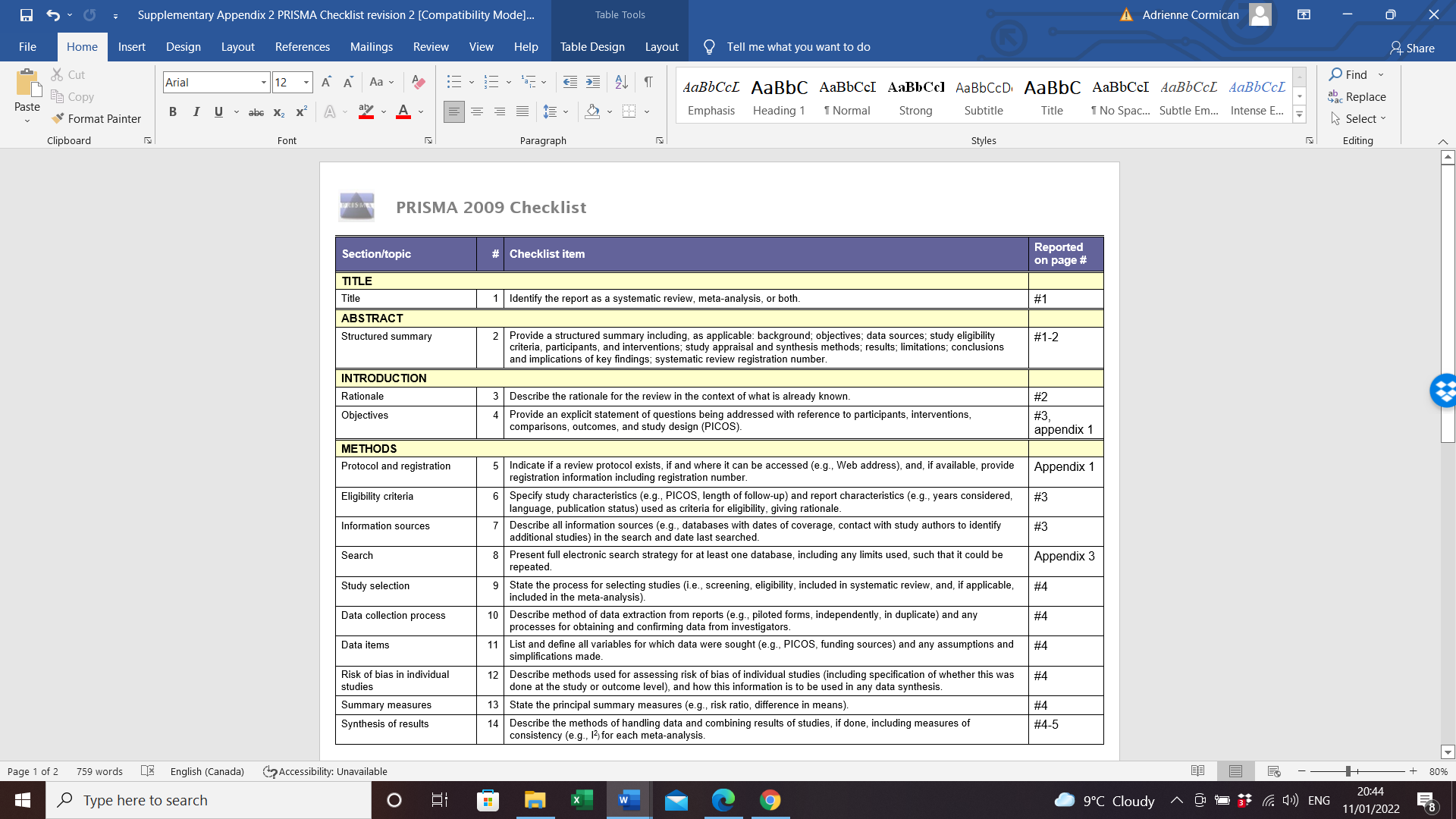


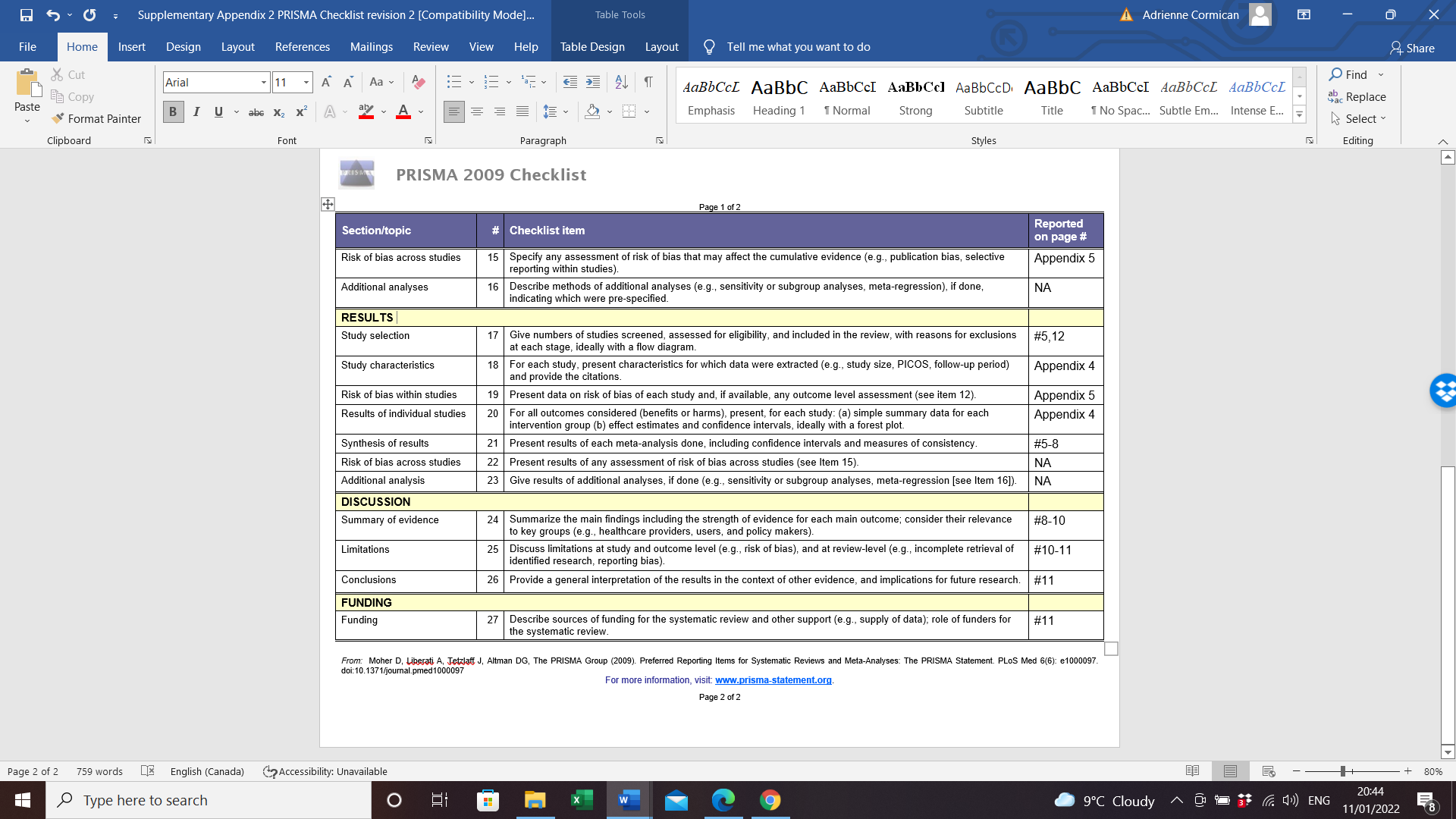


Moher D, Liberati A, Tetzlaff J, Altman DG, The PRISMA Group (2009). Preferred Reporting Items for Systematic Reviews and Meta-Analyses: The PRISMA Statement. PLoS Med 6(6): e1000097.

**Supplementary Appendix 3: Full search strategy**

**Table 1.** Search Strategy for Medline

| **Search** | **Query** |
| --- | --- |
| #1 | (MH "Guideline Adherence") |
| #2 | AB "clinical practice guideline*" OR AB "clinical guideline*" OR AB "evidence-based recommendation*" |
| #3 | (MH "Practice Guideline") |
| #4 | (MH "Evidence-Based Practice+") |
| #5 | S1 OR S2 OR S3 OR S5 |
| #6 | (MH "Stroke Rehabilitation") |
| #7 | (MH "Stroke+") |
| #8 | AB "cerebrovascular accident" OR AB "cva" OR AB stroke OR AB "stroke rehabilitation" |
| #9 | S6 OR S7 OR S8 |
| #10 | AB barrier* OR AB difficult* OR AB limitat* OR AB factor* OR AB challenge* OR AB obstacle* |
| #11 | AB facilitator* OR AB driver* OR AB enabler* OR AB motivator* |
| #12 | S10 OR S11 |
| #13 | (MH "Attitude of Health Personnel+") OR (MH "Health Knowledge, Attitudes, Practice") |
| #14 | AB view* OR AB perception* OR AB perspective* |
| #15 | S13 OR S14 |
| #16 | S5 AND S9 AND S12 AND S15 |
| Limiters | Date of Publication: 1998 - 2020  English Language  Academic Journals |

**Supplementary appendix 4**

Table 2. Data extraction table

| **Reference (Author, year, country)** | **Study design**  **Data collection**  **Analysis methods** | **Setting** | **Guideline +/- implementation intervention being discussed** | **Study aims relevant to this review** | **Participants** | **Main themes identified**  **Barriers & Facilitators** |
| --- | --- | --- | --- | --- | --- | --- |
| McCluskey et al. 2013 [49]    (Australia) | An exploratory qualitative design utilising semi-structured focus group interviewing (n=6) with the option of an individual interview (n=2)  Data was analysed using framework analysis using Theoretical Domains Framework as guiding conceptual framework. | The study was conducted with participants working in one Sydney metropolitan acute stroke unit. | The Australian Clinical Guidelines for stroke management (2010)  Interview questions focused on the delivery of evidence-based treatments recommended by guidelines, previously nominated by each discipline for improvement. Nominated areas included: UL, sensation, neglect, balance, treadmill training, swallowing, communication, education). | To identify local barriers and enablers to implementation of multiple guideline recommendations. | A total of 28 participants that represented six disciplines:  12 Medical professionals  5 OT  5 Physio  2 SLT  2 Nurses  2 Orthoptics | **Barriers:**   - Certain recommendations were too physically demanding for both therapists and patient. - Certain recommendations were time-intensive. - Patient ability (physical ability to engage, cognitive, emotional and communication impairments, medical complications, make implementation difficult, language and educational capabilities limited HCPs practice options). - Lack of availability of assessments in many languages, validated in foreign languages. Therapists were concerned language and vision problems would alter test scores. - Negative belief that a treatment may produce adverse outcomes reduced the use of some therapies (treadmill training). - Avoidance of implementing recommendations (HCPs perception that recommendations were unnecessary, less important for stroke patients, would cause embarrassment for patients). - Competing tasks, priorities, time constrains and ineffective documentation systems reduced guideline implementation. - Staff reported to forget to adhere to guidelines (especially documenting practice and blamed their business. - Limited knowledge and insufficient skills required to implement recommendations. - Other rehabilitation commitments limited guideline use among HCPs. - HCPs use their clinical reasoning to prioritize guideline interventions and their priority based on ones that produce the best patient outcome. - Resources (absence of staff, materials (written info), translators, space, time).   **Facilitators:**   - Belief that recommendations can make a difference when implemented and improve patient outcomes was enabling. - Better and improved recording systems would prompt HCPs to practice differently and improve guideline implementation. - Aware of CPGs, research and evidence, clinical protocols already available and in use, knowledge and skills to deliver interventions - HCPs that were motivated to provide a guideline intervention was a facilitator - Resources (presence of staff, translators, space, time, local protocols, necessary equipment, educational materials).   Barriers & facilitators were different across disciplines. |
| Miao et al. 2015 [50]  (Australia) | A qualitative exploratory study using semi-structured interviews,  Data was thematically analysed. | The study scope was limited to a continuum of stroke care settings across New South Wales, Australia. Participants worked in 5 acute, 5 inpatient, 7 outpatients, 3 community settings. | National Stroke Foundation Clinical Guidelines for Stroke Management 2010. | To identify speech pathologists’ perceptions and experiences of barriers and facilitators to implementing the Clinical Guidelines for Stroke Management 2010. | Speech pathologists who were required to be qualified and had worked in primary stroke care in the past 12 months and had used the National Stroke Foundations Clinical Guidelines for Stroke Management, 2010. Eight speech pathologists working across continuum of stroke care were recruited. | **Barriers:**   - Lack of guideline awareness - Lack of access, locating contextually relevant information from sizeable GPC. - Challenge in considering implementation beyond daily tasks - CPG are not highly instructive - Transferability to own context (need to understand how CPGs applied to local context) - Specificity of CPG (the breath of recommendations breath of CPGs and this flexibility allowed significant variation in practice). - Auditing criteria measuring CPG adherence was not specific & objective to accurately measure. - Lack of collaboration +/- with management, absence of key authority in co-ordinating implementation efforts, - Absence of key authority was reported to be a barrier to initiating coordinated implementation efforts. - HCP experience (novice clinician basic learning needs need to be management before CPG implementation, experienced clinicians can be potentially disadvantageous as it can be hard to change their ways,). - Lack of funding and resources leads to decrease capacity to implement CPGs. - Feasibility issues can occur when aligning services priorities when planning CPG implementation leading to prioritising some recommendations to use staff effectively. - Negotiating CPG implementation is demanding on time & resources. - HCPs wariness of CPG limitations and EBP limitations supporting them can act as a barrier.   **Facilitators:**   - Increasing knowledge and awareness of guidelines through professional development, undergraduate education or exposure to similar guidelines. - Ease navigating guidelines by specific sections relevant to settings. - Focus on implementation and monitoring implementation as a necessity through formalised team/dept meetings to review performance & plan solutions. - CPGs affirm existing knowledge - Practical local facilitators that aligned with CPGs such as screening tools, pathway documents, CPG user guides facilitating transferability of CPGs into own context. - Specificity of CPG recommendations (breath of CPGs enabled use of clinical judgement and flexibility, some felt if CPGs had more specific information about optimum therapy intensity). - Demand for change (Internal coordination of implementation, achieve change through teamwork, collaboration with management, management structure, leadership, internal collaboration with staff and patients to hold services accountable for recommended care, clinician level and allocated responsibilities varied at clinician level with senior people taking more responsibility). - Experienced clinicians facilitate implementation - Integrating recommendations into existing services/processes/routines that can support CPG implementation - Internal and individual capacities supplemented by external networking facilitated through discussion and collaboration on concrete implementation projects in peer supervision groups, special interest groups, EBP groups, working parties.   Many identifying CPG in ratifying their practice as its primary benefits, CPG supports therapists advocating for best practice and justifying new initiatives. |
| Hickey et al. 2019 [51]  (Australia) | A qualitative process evaluation embedded within a pilot cluster randomised controlled trial.  Data was collected using focus groups post implementation phase and analysed using content analysis | This study was conducted across four sites (Acute hospital settings) in Australia. | National Stroke Foundation Clinical Practice Guideline (CPG) 2010 aphasia recommendations  targeting information provision and collaborative goal setting. | To understand the nature of the impact of two tailored implementation interventions for two aphasia recommendations as perceived by the speech-language pathologists who participated in the AAIMS (The acute aphasia implementation study). | Nineteen speech-language pathologists from four sites. Eligibility criteria to participate in this study included attendance at the workshop or participation in the implementation study. | **Barriers:**   - Workshop attendance was challenging and time consuming. - Lack of electronic infrastructure, not feasible to leave electronic devices with patients. - Time limitations in acute settings led to prioritization of competing demands, short hospital admissions with a focus on discharge prevented follow up, medical focus impacted implementation. - Patient factors (aphasia severity, non-English speaking background, negative patient response/different patient priorities).   **Facilitators:**   - Workshop facilitators (accessible, tailored, face to face workshops promoting team learning, team work, team discussions, team problem solving, practical activities such as role play facilitated learning, prompted reflection and increase awareness of current practices. Increased confidence and motivation to implement CPG recommendations). - Information dissemination via workshop video, meetings with colleagues. - Audit and feedback acted as a catalyst for change, benchmarking was motivating to therapists.   Implementation facilitators - accessible and tailored resources, positive reinforcement from patient and family responses, reminders through documentation template. |
| Hadely et al. 2014 [52]  (Australia) | A 39-item online survey was used in this study.  Content analysis, descriptive and inferential statistics were used to analyse the data from the surveys.  To analyse the open-ended responses a simple content analysis (i.e. open coding, coding sheets, abstraction). | Extensive distribution of the survey using a variety of networks to target Speech pathologists who worked in stroke in public and private settings, metropolitan, rural, and remote areas in Australia were targeted.  Participants working in a continuum of stroke care settings included (27 acute settings, 30 inpatient setting, 12 outpatients, 15 community setting, 1 residential setting, 21 combination of all settings). | A whole range of CPGs were discussed as the survey left open the question about stroke CPGs.  CPGs mentioned under Q14-23 in the survey included:  -The National Stroke Foundation: Clinical Guidelines for Stroke Management (2010),  -The National Stroke Foundation 2007 edition,  -Local created CPGs which included other referenced CPGs  NZ Clinical Guidelines for Stroke Management 2010, Canadian Stroke guidelines, Scottish Intercollegiate Guideline Network 2018, United Kingdom Royal College Speech and Language Therapy Communicating Quality, United States Veterans Affairs Stroke Clinical Guidelines,  No specific CPGs recommendations were mentioned. | To describe speech pathologists’ experiences and current use of guidelines, and to identify what factors influence speech pathologists’ implementation of stroke CPGs. | Speech pathologists working around Australia in stroke rehabilitation settings who had used a stroke CPG were invited to participate N=320.  Data from 254 / 320 participants was used in the study. | **Barriers:**   - Time (insufficient time to carry out recommendations). - Work environment (organizational support required, lack of organizational priority due to competing policies, lack of resources, poor staffing, education, lack of influence/interest from others, leadership, lack of treatment space). - CPG itself (lacks high level evidence, poor information/format/design to use them, not practical, not clear, limited detail, do not add anything new to participants practice). - Therapists’ characteristics (insufficient skills to implement the interventions, lack of experience, tension between CPG and own experience, lack of trust/disagree with CPGs). - Patient characteristics (spectrum of stroke, severity of deficits, complex co-morbidities). - Lack of implementation interventions (CPG evaluation i.e. audits).   **Facilitators:**   - Work environment (influence/interest from others, workplace policies/procedures/pathways, quality improvement projects, accessibility to CPGs/resources/time, staff training, support, auditing and feedback, managers making CPG high priority) - Guideline characteristics (Clarity of information, level of evidence-base, guideline promoting client centred practice, CPG comprehensive reference list, practical recommendations that are easy to read, navigate, CPG tools (algorithms, flowcharts, summary documents). - Therapists characteristics (motivated/desire to implement CPG, having skills, experience similar to CPG recommendations). - Patient characteristics (aphasia that is not severe, using CPG as a tool to advocate for more therapy/length of stay). - Implementation strategies (dissemination, peer support, educational meetings, workshops and resources, workplace policies, multifaced interventions, stroke working parties, having a master’s degree, audit and feedback, team meetings, financial support, time, increase in staffing, external outreach visits, reminders, stronger research evidence-base, management support to sustain implementation). |
| Shrubsole et al. 2019 [53]  (Australia) | A qualitative study design using semi-structured interviews.  Data was analysed via content analysis with factors coded to the domains of the Theoretical Domains Framework. | The study was conducted with Australian speech pathologists working in aphasia rehabilitation within hospitals (acute and inpatient rehabilitation) in the Australian states of New South Wales and Queensland. These clinical settings were selected as there is routine auditing conducted at a national level in both acute and inpatient rehabilitation settings by the National Stroke Foundation. | National Stroke Foundation Clinical Guidelines for Stroke Management 2010- specific recommendations relating to 5 priority practice areas (Aphasia-friendly information provision, collaborative goal setting, timing of therapy, amount and intensity of therapy, and conversation partner training). | To explore factors (Barriers and facilitators) influencing Australian speech pathologists guideline recommended aphasia management practices in in 5 practice areas.  A secondary aim of this study was to understand the findings in the context of organisational and clinician-related factors, particularly in relation to the acute and rehabilitation settings. | Twenty speech pathologists were purposively sampled from a total of 28 participants.  20 participants were working in the following clinical settings:  8 working acute and rehabilitation setting,  5 acute only,  7 rehabilitations only. | **Barriers:**   - Environmental context and resources (lack of resources, time, competing caseload demands, physical environment (noisy), difficult to access patient and family in acute settings, difficulties in accessing staff to provide training, organizational culture that focuses on medical model of care and does not value CPGs as a priority). - Lack of time to develop appropriate resources. - Therapists’ belief about the effectiveness of the recommended practice, that patients are not appropriate for CPG recommendations, that patients will improve without such recommended interventions. - Lack of staff and patient and family expectations around recommended interventions inhibits implementation. - HCPs lack of self-confidence, self-efficacy to provide recommended interventions. - Lack of MDT leadership and engagement. - Lack of patient/family expectation/desire and a lack of understanding by patients and families about CPGs deters their use. - Lack of awareness of how to modify CPG information, of available resources, lack of familiarity with recommendations in CPG, unaware of underlying evidence for recommendation in CPG. - Lack of self-confidence in developing resources/engaging in each recommendation, in engaging/providing service to patients with severe aphasia. - Lack of perceived behavioural control due to time/caseload.   **Facilitators:**   - Environmental context & resources (access to resources, timetabling facilitates access to patients for therapy, resources such as students, therapy assistants can facilitate improved implementation of CPGs, CPG given high priority). - Therapists’ belief that patient will benefit from CPG intervention and improve their outcomes, - Social influences that can facilitate implementation - anticipated regret if recommendation is not implemented. - Having adequate knowledge about the procedural aspect of implementing recommended interventions and the theoretical knowledge behind such recommendations. - Therapists perceive that it is within their role to advocate for best-practice using CPGs. - Staff and patient/family have expectations and knowledge around recommended interventions facilitate their use. - The presence of MDT leadership, engagement, understanding facilitates CPG use. - Therapists who are aware of the need to improve practice to meet CPGs, those who have a motivated intention to improve themselves as an individual therapist, the team and the service. - Presence of self confidence in providing recommendation. - Therapists with a strong belief that the recommendations are within SLT roles and SLT need to advocate for such recommendation. |
| Jolliffe et al. 2019 [54]  (Australia) | A mixed-method explanatory sequential study which included both open and closed-ended survey questions.  Quantitative (online survey) analysis included descriptive statistics and thematic content analysis of free-test responses.  *(Responses later shaped follow-up focus group discussions)*  Qualitative (focus groups using semi-structured interviews) data analysis included thematic coding and mapped against the Theoretical Domains Framework.  Quantitative analysis was brought into qualitative dialogue.  Convenience sampling was used to recruit. | Participating organisations were two large, public funded health services providing neurorehabilitation on dedicated stroke /neurological wards as well as community neurorehabilitation services (home and centre-based contexts).  The third organisation was a smaller private practice providing community neurorehabilitation (home and centre-based contexts). | Stroke Foundation’s clinical practice guideline (Stroke Foundation, 2017).  The specific guideline recommendations referred to in this study included  1. Upper limb dose (to provide as much scheduled therapy as possible),  2. Strength (to use electrical stimulation in conjunction with motor training to overcome weakness),  3. Activity (to use constraint-induced movement therapy with eligible patients to improve arm and hand function),  4. Activity (to use mental practice in conjunction with active motor training to improve arm and hand function),  5. Activity (to use repetitive task-specific training to improve arm and hand function). | To investigate occupational therapists and physiotherapists’ perceptions of motivators and barriers to implementing and using upper limb clinical practice guideline recommendations in stroke rehabilitation. | PT and Physio responsible for delivering upper limb rehabilitation with stroke survivors at one of the three participating organisations.  N=46 participants completed the online survey (55% response rate) and 29 of these participated in one of the six focus groups.  Survey: OT n=24, Physio n=21,  Focus Group: OT n=17, Physio n=12. | **Barriers:**   - Therapists limited training or skills training in speciﬁc CPG recommended interventions. - Limited therapist skill level and confidence to select and/or complete intervention, and grade an upper limb program - Limited therapist experience in providing recommended interventions. - The complexity of intervention protocols and beliefs about intervention effectiveness. - Therapist lack confidence to apply the evidence. - Difficulty applying an intervention protocol in the “real world” (i.e. clinical practice settings. Therapists explained that ‘the real world’ does not enable best practice, with all focus groups raising this issue and all participants in agreement. - Environmental constraints (ward layout, daily ward processes and structure). - Time constraints (limited time in sessions, limited therapists’ availability) to implement CPGs. - Competing organizational priorities (discharge planning) and competing patient demands (therapy goal preferences, discharge needs). - Specific patient factors (cognitive and language impairments). - No colleague-to-colleague monitoring, no regular review of practice quality and limited accountability of therapists. - Lack of behavior regulation or monitoring of upper limb practice regarding CPG adherence. - Limited access to resources especially equipment, assessments/tools and staff to implement CPG recommendations. - Team culture and hospital policies did not always facilitate the uptake of best practice recommendations. - CPG recommendations lack specificity. - Therapist awareness of CPGs does not follow through to implementation. - Limited access to resources (i.e. equipment availability, access to assessments/ tools, staff)   **Facilitators**   - Therapists having sound knowledge of guideline recommendations and research evidence about intervention effectiveness. - Past experience with speciﬁc interventions. - Availability of required resources and an enabling workplace culture. - A “Champion therapist” with upper limb expertise and confidence that can inspire and motivate the team to deliver best practice. - Regular exposure to appropriate patients, personal experience and access to expert colleagues facilitated learning. - High organizational expectation to use guidelines in practice. - Access to training and time to put training into practice. - Access to resources (equipment, assessments, tools). - Reinforcement for using recommended interventions such as if it was commonplace, observed interventions effectiveness, patient enjoyed it. - Therapists more likely to use or continue using an intervention if they have observed the intervention’s effectiveness, irrespective of its level of research evidence. - Access to group therapy/programs (i.e., patient’s involvement and/or access to group therapy). - Supportive team and/or management including mentoring and supervision. - Collaborative approach to upper limb management (i.e., OTs and Physio’s working together). - Access to family members and therapy assistants for intervention involvement. - Motivated clients. |
| Bayley et al. 2013 [55]  (Canada) | A pilot implementation study with a “local facilitator” to promote the 6-month implementation.  Focus groups done at completion were analyzed thematically for barriers by two raters. | Five hospital centres providing inpatient stroke rehabilitation in five Canadian cities, selected to represent various geographic locations, hospital affiliations (academic or non-academic) and sizes. | The Stroke Canada Optimization of Rehabilitation (SCORE) by Evidence project developed evidence-based recommendations (EBRs) for arm and leg rehabilitation after stroke. | The specific objective of this study is to describe the barriers to the introduction of the EBRs for stroke rehabilitation experienced by nurses, occupational therapists, physical therapists, physicians and hospital managers and to discuss the implications for future attempts and increasing evidence use in rehabilitation. | 79 rehabilitation professionals (23 occupational therapists, 17 physical therapists, 23 nurses and 16 directors/managers) participated in 21 focus groups of three to six participants each. | **Barriers:**   - Lack of time to implement, set, up deliver (lack of time to read recommendations, lack of time to attend meetings, lack of time to set up some recommended interventions). - Staffing issues (inadequate staffing, high turnover, lack of support for new staff, need for management support). - Training/education (difficulty understanding terminology education in recommendations, insufficient training, need for hands on demonstrations rather than didactic lectures, lack of trainers). - Therapy selection and prioritization (patients too severely impaired or lacked tolerance for all recommendations). - Research evidence supporting recommendations influencing use of recommendations. - Resources (limited treatment space, equipment availability, difficulty locating equipment, inappropriate equipment). - Team functioning/ communication (insufficient MDT collaboration, lack of implementation discussions and planning, lack of management support, lack of teamwork, difficulty organizing meetings and continued contact, lack of expertise to demonstrate how to implement recommendations to other team members, lack of authority/confidence to teach other members).   These varied by OT, PT, Nursing and manager responses. |
| Munce et al. 2017 [56]  (Canada) | A qualitative descriptive methodology using semi-structured telephone focus groups lasing approximately 45-60 minutes.  Data was analysed using thematic analysis and framework analysis using the Clinical Practice Guideline Framework for Improvement. | The study was conducted with participants representing 11 sites (rehabilitation centres) that took part in the cluster randomized implementation trial (SCORE-IT), 6 sites from the facilitated KT arm and 5 sites from the passive KT arm from western, central and eastern Canada. | Recommendations in the SCORE guidelines (Canadian Stroke Best Practice Guidelines) targeting upper limb and lower limb function, mobility and postural control. | To understand the factors influencing the implementation of recommendations and KT interventions from the perspectives of nurses, occupational therapists and physical therapists and clinical managers following completion of the trial. | Focus groups were conducted with 33 participants (11 therapists including both occupational therapists and physiotherapists, 11 nurses, 11 clinical managers). | **Barriers:**   - Lack of continued facilitation (enabling change for both arms of trial impacted on continuity and sustainability of KT interventions during and after KT trial). - Lack of agreement with the intervention (staff perceived interventions as not practical or clear, information was too vague) - Some interventions were too time-consuming discouraging implementation. - Lack of familiarity with recommendations discouraged use. - Lower volume of patients was associated with fewer opportunities to practice implementing interventions. - Environmental barriers (time pressures due to competing initiatives/roles/ responsibilities, insufficient staff, high turnover, lack of space and equipment, - Lack of active support from senior management and a lack of leadership.   **Facilitators:**   - Facilitation via champions, leadership or local interested leaders enhances intervention uptake, facilitators provide support, motivation and continuity in the face of high staff turnover. - Agreement with the intervention because they were practical to follow, clear and supported implementation of guidelines. - Familiarity with CPG recommendations - Inclusion of evidence-base supporting recommendations underscored their importance. - Regular prompts (pocket cards, posters).   Team communication and interdisciplinary collaboration fostered via educational interventions in both arms of trial increased staff’s understanding “why” behind CPG recommendations. |
| Fisher 2014 [57]  (Canada) | A sequential exploratory mixed method,  Design using focus groups, chart audits and survey.  Nil analysis method stated for qualitative data.  Data from the survey will be analysed using descriptive statistics.  Quantitative chart audit data: not relevant to the present review, i.e., did not investigate facilitators and barriers to CPG implementation. | The study was conducted in two hospital settings: a rehabilitation setting and a mixed acute care and rehabilitation setting (stroke rehabilitation units) in Ontario, Canada. | The Clinical Practice Guidelines for the Urinary Continence Care of Stroke Survivors in Acute and Rehabilitation Settings.  Only 3 recommendations were selected for implementation on each site. | The study objectives were to identify key factors that may be barriers or facilitators to urinary management practice | Between 25-30 participants (clinical staff nurses) attended focus Groups. It does not give exact number or breakdown of participant characteristics.  Clinical staff nurses completed the survey:  N=40 pre-pilot implementation,  N=20 post-pilot implementation. | **Barriers:**   - Lack of adequate resources (staffing, inadequate facilitates, insufficient time). - Attitudes (cultural and personal beliefs regarding continence care, low priorities regarding continence care, resistance to practice change, belief that continence challenges are acceptable), - Lack of continuity of continence care (absence of clinical practice guidelines lack of interprofessional communication and staff turnover).   **Facilitators:**   - Best practice guideline champions to advocate and support nurses and other health professions to use the guidelines in rounds, - Best practice guidelines (clear, concise, easy to understand, inclusion of clinical practice guidelines and/or information sheets in educational and patient information packages). - Structures supporting guideline implementation (interprofessional teams, policies supporting evidence-based practice changes, expert clinics and multidisciplinary processes).   **Strategies to assist with implementation:**   - Identity and capitalize upon practice champions using existing systems in place or develop new champions through education and outreach. - Implement educational program: a multi-tiered educational program targeting different health professionals, patients and carers using high-quality presentation materials and self-learning packages. Make these educational materials available to patients and caregivers to increase their awareness of post-stroke continence challenges.   Implement practice changes incrementally so changes are manageable by nurses. |
| Donnellan et al. 2013 [58]  (Ireland) | A qualitative action research design using 3 stages to guide this study.  1. Literature review and stakeholder focus group/interview  2, Exploratory focus groups using semi-structured interview schedule.  3. Dissemination of findings and application in planning and development.  Thematic analysis was carried out on data collected.  (stage 1 and 3 are not relevant to the present review) | Stage 2 of this study (exploratory focus groups) was conducted with acute hospitals in 3 different health regions (South-East, Midlands, Dublin incorporating urban and regional hospitals. | Irish National Clinical guidelines and recommendations for the care of people with stroke and transient ischemic attack, revised version 2010. | 1. To examine stakeholders and HCPs views and opinions on implementing national stroke guidelines.  2. To identify factors that act as barriers and facilitators to implementation of guidelines. | Ten participants were involved in stakeholder focus group (4 Consultant Physician, 1 advance nurse practitioner, 1 public health nurse, 1 Physio, 1 Speech and language therapist, 2 social workers)  Thirty-six Allied health professionals  took part in hospital focus groups  (7 OT, 8 Physio, 2 Dietician, 0 SW, 5 SLT, 5 Clinical Nurse Manager, 1 staff nurse, 0 clinical nurse specialist, 0 advance nurse practitioner).  Medical professionals (5 stroke consultants, 2 specialist registrars). | **Barriers:**   - Inadequate resources (lack of time, lack of designated stroke staffing, lack of appropriate equipment). - Guideline characteristics (non-specific content, lack clarity, prohibitive size, ease of access, time to read them, acute clinical focus (over-representation of acute), lack of community-based rehabilitation services, inadequate fragmented dissemination). - Insufficient training /education: inadequate organized dissemination of guidelines, lack of managerial support, frequency of access to training.   **Facilitators:**   - Resources (protected time for stroke services, dedicated staff with special interests, dedicated stroke units, adequate and appropriate equipment and technology). - Guideline characteristics (user-friendly guidelines, localized guidelines that are relevant at local level). - Guidelines act as a tool for advocating for change and better services, auditing of services and consolidating knowledge. - Advocates (managerial support, positive organizational culture, time allocated to training, key individuals with special interest in stroke care/guidelines located within clinical settings to support and promote guidelines and drive improvements). - HCPs positive attitudes towards CPGs, valuable as a platform to set standards to aspire to.   Educational workshops/study days that drew on guidelines were considered beneficial in implementation. |
| Donohue et al. 2014 [59]  (Ireland) | This observational cross-sectional country wide study utilised a self-completing original postal or an online survey sent to Physio’s working in 31 acute hospitals  Data was analysed using descriptive statistics | A list of 33 acute hospitals involved in the HSE National Clinical Programme for Stroke was obtained for recruitment.  2 out of 33 hospitals did not fit the inclusion criteria due to absence of acute stroke physiotherapy service. | Irish Clinical Guidelines for Stroke 2009 related to assessment and rehab (10 CPG recommendations). | To identify the degree to which Senior Physiotherapists in acute stroke care adhered to clinical guidelines and to explore the barriers to adherence experienced by these physiotherapists. | Twenty-three out of thirty-one senior Physio’s responsible for the acute stroke physiotherapy service invited to participate responded. | **Barriers:**   - Poor staffing - Time constraints - organizational barriers such as underdeveloped stroke service (nil weekend PT service therefore weekend admissions, delayed physiotherapy referrals). - Lack of coordinated stroke services |
| Hafsteinsdóttir et al. 2013[60]  (Netherlands) | A four-phase cross sectional design using self-developed questionnaire.  Data was analysed using descriptive and inferential statistics. | This study was conducted in four Dutch health-care facilities: a general hospital, a university hospital, rehabilitation centre, and a nursing home. | Clinical Nursing Rehabilitation Stroke (CNRS) -guideline (Hafsteindottir et al 2009) which  includes 243 recommendations.  Each week within the four-week phase, new recommendations were introduced:  Phase 1: Theoretical background of rehabilitation (20 recommendations), mobility and ADL (59)  Phase 2: Nutrition (19), Swallowing (14) dehydration (12),  Phase 3: Cognitive problems (19), communication (20), depression (21)  Phase 4: falls (18), sexuality (20), patient education (21) | To investigate the feasibility of the use of the draft version of the CNRS-guideline (Hafsteindottir et al 2009) in the daily care of patients with stroke by: describing the views and attitudes of the nurses using it, measuring if the attitude towards the guideline was associated with the perceived innovation characteristics of the guideline; if the attitude towards the guideline was associated with the adoption of the guideline and how often the recommendations were used. | Thirty participants were nurses who cared for the selected patients whose data was used. Twenty out of the 30 worked on an acute stroke unit in a general hospital, four worked in a university hospital and the nursing home, two nurses worked on the rehabilitation centre. | **Barriers:**   - Therapists’ characteristics: attitude, belief that CNRS are not relevant for different phases of stroke, lack of knowledge and lack of skill of recommendations. - Guideline characteristics: CNRS-guidelines overlapped with other professionals’ roles, they were found to be not practical, too complex, too global and need to be linked to clinical practice, not relevant for different phases of stroke and time consuming. - Organisational characteristics: lack of coordination with other professionals, lack of training and skill development, lack of management support of CPG implementation, lack of time and more staff training in skills needed.   **Facilitators:**   - If therapists have a positive attitude towards CPGs. - CNRS were reported to be practical, pocket version would be a practical aid, written clearly, easy to apply and integrate, easy to use in supporting patients. - Management support in CPG implementation, time to implement them and to become a routine in daily care, practical pocket version, - HCPs could see the benefits of using them-give more insight into patient’s ability,   Patient benefits: support in improving patient self-efficacy and motivate pts to participant in physical training. |
| Otterman et al. 2012 [61]  (Netherlands) | A descriptive country wide cross-sectional web-based self-developed survey was used.  Data was analysed using descriptive statistics.  The perceive barriers and facilitators were calculated by counting and ranking frequencies. | The study was conducted with physiotherapists from all Dutch hospitals with an inpatient neurology department. | Dutch CPGPS (Clinical practice guideline for  physical therapy in patients with stroke) (2004). Recommendations: out of bed within 24 hours and augmented exercise therapy time; a minimum dose of 2 times x 20 minutes of exercise therapy. | To identify the perceived barriers to and facilitators for guideline adherence as reported by physical therapists working on acute hospital stroke units. | Participants included physiotherapists who provide treatment to patients in the acute poststroke phase and were identified by their manager. N=91. | **Barriers:**   - Physiotherapists that have not read the CPGPS thoroughly. - CPGPS recommendations that do not fit in with working methods in routine practice. - Adherence to CPGPS is time consuming, no financial compensation for adhering to guideline. - The applicability of the CPGPS acts a barrier as layout is not suitable for practice use. - Physiotherapists negative beliefs/attitudes about guidelines i.e. recommendations are not correct.   **Facilitators:**   - The flexibility of CPGPS leave enough room for physiotherapists to draw their own conclusions and take the patients preferences into account. - The layout of the CPGPS makes it suitable for practical use, recommendations are clearly formulated. - Sufficient knowledge and experience with the CPGPS lead to positive beliefs and attitudes by physiotherapists, no reluctance to adhere to the CPGPS,   Therapists having read the guidelines and adequate knowledge about the CPGPS to facilitate the application of it. |
| Mudge et al. 2017 [62]  (New Zealand) (NZ) | A qualitative descriptive methodology using semi-structured interviews lasting approximately 1 hour.  Content analysis with constant comparative methods | The study was conducted with NZ registered therapists, working in one of two hospitals, treating at least 10 patients with stroke in the previous year. | National NZ Clinical Guidelines for Stroke Management (2010)  Participants were given a list of guideline topics which included a summary of the guideline recommendations for each topic and asked to choose at least 2 topics to discuss. | (1) Seek perceptions of the NZ stroke guidelines in terms of utility and feasibility.  (2) Identify barriers and facilitators to NZ stroke CPG implementation. | N=11  7 OT  4 Physiotherapist  All therapists were working with patients with stroke in either an acute or inpatient rehabilitation setting. | **Barriers:**   - CPGs are said to be non-specific including complex recommendations and “overwhelming” in size. - HCPs mistrust with lower graded supporting evidence and therefore were viewed as less important, less useful and consequently implemented less frequently. - The need to fulfil policy requirements and meet the District Health Board (DHB) expectations drove clinical practice. This was viewed as a stronger influence than the NZ stroke guidelines. - A lack of processes/protocols to influence practice using CPGs. - Therapists perceived value of CPGs was raised as a potential barrier. - Lack of funding for stroke-related resources (beds, staff) required to implement CPGs. - Lack of suitable equipment in working order. - Staff prioritisations of patients and recommendations was said to affect which recommendations were implemented. - Time was frequently mentioned as a barrier to guideline implementation. Heavy caseloads and limited working hours led to lack of time with each patient resulting in poor CPG implementation. - A lack of patient and family engagement or buy-in to recommendations and their goals not aligned with CPG recommendations. - Family expectations can act as a barrier in that families may believe the patient needs rest rather than active rehabilitation. - Therapists lack of knowledge, experience, education and training precludes the implementation of certain CPG recommendations. - Application for formal courses is a difficult process and was said to impact on therapist’s knowledge of CPGs recommendation. - Certain CPG recommendations were considered difficult to implement unless the therapists were highly skilled. - Different scopes of practice between OTs and PTs leading to application of different recommendations to different disciplines. Some recommendations were neglected due to uncertainty regards whose professional role it was to implement. - OT models of practice and beliefs were the main influencers on their clinical practice, which were not always perceived to be congruent with the NZ stroke guidelines.   **Facilitators:**   - GPCs could be improvement by making recommendations clear, specific with suggestions of incorporating prioritized time frames for recommendation implementation. - A condensed version of the NZ stroke guidelines with both specific allied health and setting-specific sections would make them easier to consult and implement. - CPGs were perceived to be useful for provision of evidence. - CPGs recommendations that were congruent with therapists practice models. - Increase bed capacity and introduce slow stream rehabilitation beds. - A larger team of professionals could allow them to spend more time with each patient. - Sharing responsibilities with therapy assistants and colleagues, working collaboratively to make practice more efficient and address barriers to implementing CPGs. - On-going training for therapists to learn about complex therapies recommended in CPGs. - Protected time to read the NZ stroke guidelines. - Patient and effective family buy-in and engagement in rehabilitation. - Professional courses that teach complex skills and provided therapists with knowledge about CPGs and endorsed their use.   Reminders and meetings about CPGs were thought to increase therapist’s awareness. |
| Clarke et al. 2018 [63]  (United Kingdom) | Mixed-methods case-study evaluation,  (triangulation design) using modified process mapping, non-participant observations of service organisation and therapy delivery, documentary analysis and semi-structured interviews lasting 1 hour with 15-20 staff per unit.  Data was analysed descriptive and inferential statistics, documentary analysis and framework analysis approach.  Observation data: not relevant to the present review, i.e., did not investigate facilitators and barriers to CPG implementation from HCP perspective | This study was conducted across 8 UK NHS stroke units purposively sampled to include a mix of hyperacute, acute and rehabilitation units with higher and lower national audit ratings for therapy performance. | Royal College of Physicians National Stroke clinical practice guidelines 2016, recommends patients should ‘accumulate at least 45minutes of each appropriate therapy every day at a  frequency that enables them to meet their rehabilitation goals’ (p. 25). | To develop an in-depth understanding of therapy provision in  stroke units in England, including how clinical guideline recommendations are interpreted and  implemented by therapists, and experienced by  patients and their carers. | Observations of the following took place: Stroke patients n=77,  Carers n=53 197 stroke unit staff n=197,  Participants involved in the  semi-structured interviews included:  Stroke patients n=49, Carers n=50,  Stroke unit staff n=131.  Representation of different disciplines included:  physiotherapy n=40,  occupational therapy n=30,  Speech and Language Therapy n=30,  Generic therapy assistant n=8,  Nurse n=10,  Physician n=7,  Non-clinical manager n=8. | **Barriers:**   - Time spent on information exchange that is not therapy focused (daily handovers, board rounds) - Time spend on non-patient contact activity (planning therapy, documenting therapy provided, discharge planning, ordering equipment and transport, developing patient and family/carer training and information packages, supervision, training staff, duplication of documentation in the form of SSNAP, internal audit records). - Poor staffing levels and restricted service structure (working times, nil 7-day service). - Patient Factors (patients’ condition e.g. clinical instability, post-stroke fatigue, cognitive deficits, concurrent medical illness), patients’ physical readiness and availability to participate in therapy). - HCPs prioritization of tasks can impede guideline implementation. - Therapists’ limited knowledge of the evidence behind CPG or contradictory beliefs about the evidence of dosage and the CPG recommendation. - Influence of external audit of stroke services – unsure about what to record, poor awareness of the SSNAP audit information and how to access it. - Group therapy that is not therapeutic but done just to meet the 45-minute target.   **Facilitators:**   - Increased/appropriate/full staffing “the more of you, the more time you got”. - Shorter sessions repeated throughout the day for fatiguing patients or those who can’t concentrate to facilitate more therapy - Better communication with nursing staff improves patient’s readiness for therapy. - Working to the SSNAP standard gives staff a target to work towards and as a benchmarking approach facilitates implementation.   Use of time tables to prevent clashing with other therapists and wasting time and act as communication source for care staff. |
| Bjartmarz et al. 2017 [64]  (Iceland) | Sequential explorative mixed-method design including pre-test post-test measures and focus group interviews.  Quantitative data: Descriptive & inferential statistics,  Qualitative data: Content analysis  Quantitative and Qualitative data integrated after analysis. | The study was conducted at neurology and rehabilitation wards of a university hospital in Iceland. | A Stroke Nursing Guideline (SNG) comprised of 23 recommendations focusing on assessment and therapeutic interventions targeting ADLs, mobility and falls (14 recommendations), pain/shoulder pain (3 recommendations), depression (3 recommendations), patient education (2 recommendations), discharge planning (1 recommendation).  Over 9 months, the SNG was implemented using the following evidence-based implementation strategies: education, training, opinion leaders, posters, reminders. | What are the nurses’ and auxiliary nurses’ view on the accept-ability of using the SNG in supporting the provision of daily nursing care?  What are the nurses’ and auxiliary nurses’ views on barriers and facilitators to implementing and embedding the SNG within routine daily nursing care? | Thirty-three nursing staff responded to the pre-test questionnaires, whereas 25 responded to the post-test questionnaires (18 nurses/15 nursing auxiliaries/pre-test and 13nurses/12 nursing auxiliaries/post-test).  Sixteen nurses and auxiliary nurses (N= 8 each group, respectively) took part in three focus group interviews.  Those who completed focus groups had already completed survey. | **Barriers:**   - Severe organizational and budgetary restrictions were taking place.   **Facilitators:**   - HCPs had knowledge of CPG content, used it and found it practical, concise and easy to use. CPG layout was convenient, pictures were illustrative and instructive for patients, family members and nursing staff. - HCPs reported to see the benefits of using CPG in providing consistency and improved patient care. - CPG provided a clear definition of nursing rehabilitation and enabled integration of essential rehabilitation components into daily nursing care. - Good, consistent and enhanced staff, patient and family education including teaching material. - Nursing staff reported a positive experience in implementing CPGs. - Nursing staff report coherent and consistent leadership was provided to aid implementation.   Of 37 items on screening and application of key interventions in stroke care, was improved in 23 items after implementation. |
| Jolliffe et al 2020 [65]  (Australia) | Non-randomised three-arm cluster-controlled feasibility study with assessment at three time points including medical file audit and post intervention survey and focus group.  Quantitative data was analysed using descriptive and inferential statistics.  Qualitative data: thematic analysis.  Quantitative analysis was brought into qualitative dialogue. | This study was conducted across 6 sites: 3 inpatient and 3 outpatient services purposively sampled to include a mixture of inpatient and outpatient services in Melbourne, Australia. | Upper limb rehabilitation related guidelines from the Stroke Foundation Clinical Guidelines for Stroke management 2017.  Implementation intervention focused on key recommendations: task-specific motor training, functional electrical stimulation, upper limb programmes, modified constraint induced movement therapy. | The aim of this study was to test the feasibility and potential efficacy of two tailored implementation packages for improving adherence to upper limb stroke rehabilitation guidelines and to understand the acceptance from the therapists’ perspective. | 25 Occupational therapists and 4 physiotherapists.  11 therapists were working in outpatient setting and the other 18 in an inpatient setting.  18 therapists completed postintervention survey and 6 therapists in the focus group.  55 patient participants were recruited. 19 were recruited from an outpatient setting, and 36 from an inpatient setting. | **Barriers;**   - Less protected time for participants in self-directed implementation package resulting in therapists not having adequate time to review all resources. - Less structured environment, less face-to-face sessions with hands on demonstrations in self-directed implementation package which would have assisted with greater uptake of guidelines.   **Facilitators:**   - Facilitator-mediated implementation package was feasible and acceptable. - Implementation package resources were helpful, relevant and assisted in provision of upper limb guideline implementation. - Tailored resources in facilitator-mediated group were valuable and accessible. - Implementation package resource availability and equipment were invaluable in time efficiency for setting up patient programmes and prioritizing upper limb rehab. - Implementation package skilled behaviour monitoring incentivized evidence-based practice. - Structured learning environment, direct mentorship & modelling prioritized and facilitated optimal learning in participants in the facilitator-mediated group. - Implementation package education and regular audit and feedback were helpful. - Implementation programme stimulated team discussions and plans for other resources required to continue uptake of guidelines. - Resources were easy to access and navigate. - Study participation increased skill, knowledge and confidence. - Auditing and feedback sessions motivated and encouraged therapists.   Overall, involvement in the study had changed therapists’ practice. |
| Connell et al 2014 [66]  (Canada) | Cross-sectional study design via semi structured interviews.  Data was analysed using directed content analysis using three frameworks from implementation science. | 8 sites (7 regional hospitals and 1 rehabilitation center) in British Columbia, Canada. | Canadian Best Practice Recommendations for Stroke Care (update, 2010) recommendation to “provide a graded repetitive arm supplementary program for patients to increase activity on ward and at home”. | The aim of this study:  - Explore the processes that therapists’ report was involved in implementing GRASP in practice.  -Explore therapists’ experience of using GRASP in clinical practice and how this adheres to intervention components outlined within GRASP guideline.  -Use a taxonomy of factors influencing implementation to explain the research findings. | This study interviewed:  13 Occupational Therapists,  5 Physiotherapists, 2 rehabilitation assistants. | **Barriers:**   - Therapists’ knowledge and beliefs: Concerns about quality of exercises in self-directed practice. - Acquiring and organizing necessary equipment was most challenging process in implementing GRASP. - All components of GRASP intervention were modified when implemented in practice - Completed by individual therapist level but not at team/departmental level.   **Facilitators:**   - Evidence strength & quality: well supported evidence-base for GRASP program. - Good design, quality and packaging: GRASP program handout is clear, well laid out. - Leadership engagement: Key individuals at each site to initiate/support GRASP implementation - Rehabilitation assistants go over the GRASP program a few times until they can complete without assistance. - Therapists positive first impressions from using GRASP. - Access to knowledge and information: Free online availability of GRASP materials.   Relative advantage: GRASP provided more time efficient way of providing exercises to patients. |
| Cahill et al 2021 [67]  (Australia) | Descriptive qualitative study design involving pre-implementation questionnaires, focus groups and separate interviews for those unable to attend focus group.  Data was analysed using inductive approach (thematic analysis) and deductive approach using implementation theory (the theoretical domains framework and Normalisation Process Theory). | The study was conducted across 8 sites (6 public and 2 private organisations) across Victoria and New South Wales Australia.  All sites were tertiary health services. | Stroke Foundation Clinical guidelines for Stroke management 2020 recommend standard assessment and sensory-specific treatment for somatosensory loss | The aim of the study was to explore barriers and facilitators and any other factors influencing implementation of somatosensory guidelines (somatosensory assessment and intervention) in practice to provide an explanation of underlying mechanisms that enhance or inhibit such implementation. | 87 therapists participated in focus groups and interviews across all sites.  69 Physiotherapists  18 Occupational Therapists | **Barriers:**   - Therapists’ self identified lack of knowledge (procedure knowledge about how to complete assessment / intervention), lack of skill and confidence. - Therapists expressed negative emotions (concern, guilt, frustration, fear) relating to lack of knowledge, skill and confidence. - Lack of confidence leads them to deprioritise this area of practice. - Lack of opportunities for development and skill consolidation after upskilling in evidence-based rehab. - Patients poor understanding of sensory deficits and sensory rehabilitation not perceived as a priority and not a patient goal to work towards. - Patient communication or cognitive impairment and also need somatosensory rehabilitation. - Organisational barriers & system pressures by competing demands (discharges) and reduced opportunities to provide somatosensory rehabilitation. - Lack of resources: equipment, disorganized tools, quiet spaces, - Overlapping professional roles - physiotherapy defer professional responsibility to complete somatosensory rehabilitation to Occupational Therapists. Occupational Therapists uncomfortable with “expert” label. - Implementation is left up to individual therapists   **Facilitators:**   - Having correct equipment would improve practice, confidence, skill development in delivering somatosensory rehabilitation. - Organisational identity / brand enables therapists at organisations which aspire to provide evidence-based practice. - Interpersonal processes where colleagues supported practice change. - Team training on somatosensory intervention so effect change at team level. - Patients who were well informed about somatosensory treatment. - Therapists had positive views about effectiveness of somatosensory rehabilitation and observing the direct benefit of it with patients. - Belief in the evidence base for Somatosensory rehabilitation and helped support the anticipated time required to change practice. |
| Inness et al 2022 [68]  (Canada) | A descriptive qualitative study design using interviews and focus groups.  Qualitative content analysis using both deductive and inductive coding.  First, deductive coding was mapped to the domains of the Theoretical Domains Framework.  2^nd^, inductive coding to identify overarching themes across the data set. | Purposively recruited from 4 stroke rehabilitation settings  in Ontario, Canada, who were already participating in a separate multi-site trial on  implementing an Aerobic intervention. | Aerobic Exercise Recommendations to optimise best practice in care after stroke (AEROBICS) best practice guideline (updated 2013).  The supervised Aerobic intervention was a group program for 20-30 minutes,  provided up to 3 times per week. | The main objective was to identify key barriers, enablers, and implementation considerations  to inform the development of a clinical implementation toolkit  to support uptake of Aerobic intervention in stroke rehabilitation settings. | 7 managers who had experience implementing the Aerobic intervention in their setting, 24 were health care professions providing direct care and aerobic programme intervention.  6 clinician focus groups:  24 physiotherapists  1 Occupational Therapist  5 Rehabilitation assistants,  1 nurse  1 Physician | **Barriers:**   - Prioritizing the provision of aerobic intervention due to resource constraint. - Aerobic exercise viewed low priority/long term goal versus patient goal to improve function in inpatient stroke rehabilitation. - Competing demands limiting provision of aerobic exercise as standard of care. - Therapists’ safety concerns implementing aerobic exercise with patients.   **Facilitators:**   - Participants acknowledge Aerobic intervention as best practice. - Team roles (Physician’s, nurses, rehab assistants, - Physician’s’ roles in supporting pre-participating screening or referring patient for tests when warranted before aerobic intervention. - Nurses play key role in providing information of medical status pre-exercise screening. - Rehab assistant’s important supportive role in patient set-up and monitoring during exercise. - Formal and informal leadership roles were enablers - Managers that promoted culture of learning and supporting staff involvement and securing necessary resources - Leaders within practice environment (local champions / opinion leaders) were seen to facilitate and influence engagement of other staff. - Need for formal implementation leaders with dedicated time to provide support, organize staff education, engage teams in planning processes and leading implementation strategies - Team engagement in planning and implementation process. - Strategies that increase self-efficacy and capability in providing the intervention: education, skill development, clinical experience, using clinical tools to support clinic-decision making. - Access to experts in aerobic exercise locally or in other settings. - Supportive scheduling processes and team collaboration to share space, equipment and assistants. - Development of forms, referral processes. |
| Auchestaetter et al 2016  [69]  (Canada) | Cross-sectional study.  Descriptive statistics were used.  The closed-ended survey questions identified few barriers or facilitators that  were common to the majority of respondents.  Open-ended questions were thematically analysed. | Therapists who worked in the area of stroke  across the continuum of care, geographical locations,  and in both public and private settings were targeted across Canada. | The  American Stroke Association clinical practice guideline for the management of Stroke rehabilitation (2010) recommend FES for gait and upper extremity  training, including shoulder subluxation,  in clients with impaired muscle contraction.  Two Canadian clinical practice guidelines also recommend FES for stroke  Care (Canadian Partnership for Stroke Recovery Intervention 2012, Evidence-based review of Stroke rehabilitation, 2014). | To identify the barriers and facilitators  influencing the use of FES. | 298 Physical therapists participated.  244 responses in open ended question received for to facilitators and 221 responses received for barriers. | **Barriers:**   - Lack of training in FES. - Lack of resources (time, equipment, access to devices). - Limited support from suppliers of FES devices, colleagues not using FES and high turnover staff. - Lack of knowledge, training and expertise, - Perception of FES being inappropriate for certain patients with client factors such as dementia, spasticity, poor skin quality, frailty, poor sensation, presence of a pacemaker, acute injury - Therapists’ preference to use other treatment options   **Facilitators:**   - Hands-on training and experience via continuing education courses peer teaching, train the trainer programs. - Positive effects that are observed from FES with patients. - Access to resources (time, equipment, funds, support) to enable application of FES. - Comfortable and confident in applying FES. - Existence of high-quality evidence for the efficacy of FES- therapists being aware of this evidence and clinical guidelines. - Client characteristics (being motivated, able to work independently, supportive family, having equipment) that increase the likelihood of adherence and transition to the home environment. - Identifying therapeutic goals that can be appropriately addressed with FES. - Support from colleagues in leadership positions and ed to creative methods (FES group sessions). |
| Chang et al 2018 [70]  (Australia) | Quantitative cross-sectional study design using web-based survey.  Data was analysed using descriptive statistics.  Survey statements corresponded to The Theoretical Domains Framework (TDF) using a liker scale accompanied with illustrative  quotes from the open-ended responses on barriers and  facilitators.  Open-ended questions were analysed using conventional content analysis before being mapped onto the TDF categories. | Clinicians who worked in the area of stroke,  geographical locations,  and in both public and private settings were targeted  using a variety of strategically chosen mailing lists, social media and websites (e.g., Stroke  Foundation). | The provision of communication partner training (CPT) to unfamiliar and familiar Communication partners (CPs)  of people with aphasia is recommended in  national clinical practice guidelines (Stroke Foundation 2017). | An aim of this study was to investigate Australian speech pathologists’ factors influencing the implementation of CPT programmes in practice | 122 speech pathologists were surveyed.  32 speech pathologists worked in acute services,  42 in Inpatient rehabilitation  48 in Outpatient  rehabilitation/community | **Barriers:**   - Lack of systems for monitoring whether CPT was provided and workplace policies / procedures that facilitated CPT. - No guidelines or structure from management to support CPT to occur in a systematic way. - Lack of opportunities for formal training and skills to provide CPT. - Negative belief about consequences: majority do not perceive clear benefits of CPT. - Lack of social influences: not talked about as an essential practice, lack of engagement of CPs as they don’t value it. - lack of sufficient resources. - CPT is often forgotten about or not a priority due to competing demands. - Lack of confidence in providing CPT. - Lack of clear structure / explicit instructions in how to provide best practice CPT in research. - Patient-related factor barriers: busy timetable in acute rehab, too tired to participate. - Limited Organisational profile of speech pathologists as communication experts.   **Facilitators:**   - Blocking out sessions for this goal, regular training slots for staff who are CPs, procedures in place to make accountable for providing training - Receiving positive feedback, recognition and support from clients, colleagues for doing CPT. - CPT is compatible with regular clinical practice. - CPT is identified as part of their role. - Buy in from patients that CPT will help them. - Speech pathologists found CPT rewarding. - Individually, speech pathologists are making it their personal high priority goal to provide CPT. - Belief in the strong evidence for CPT. - Confidence in providing CPT boosted by clinical experience. - Organizations that provide sufficient resources and willing to respond to challenges in providing CPT. |

**Supplementary appendix 5- Mixed method appraisal tool**


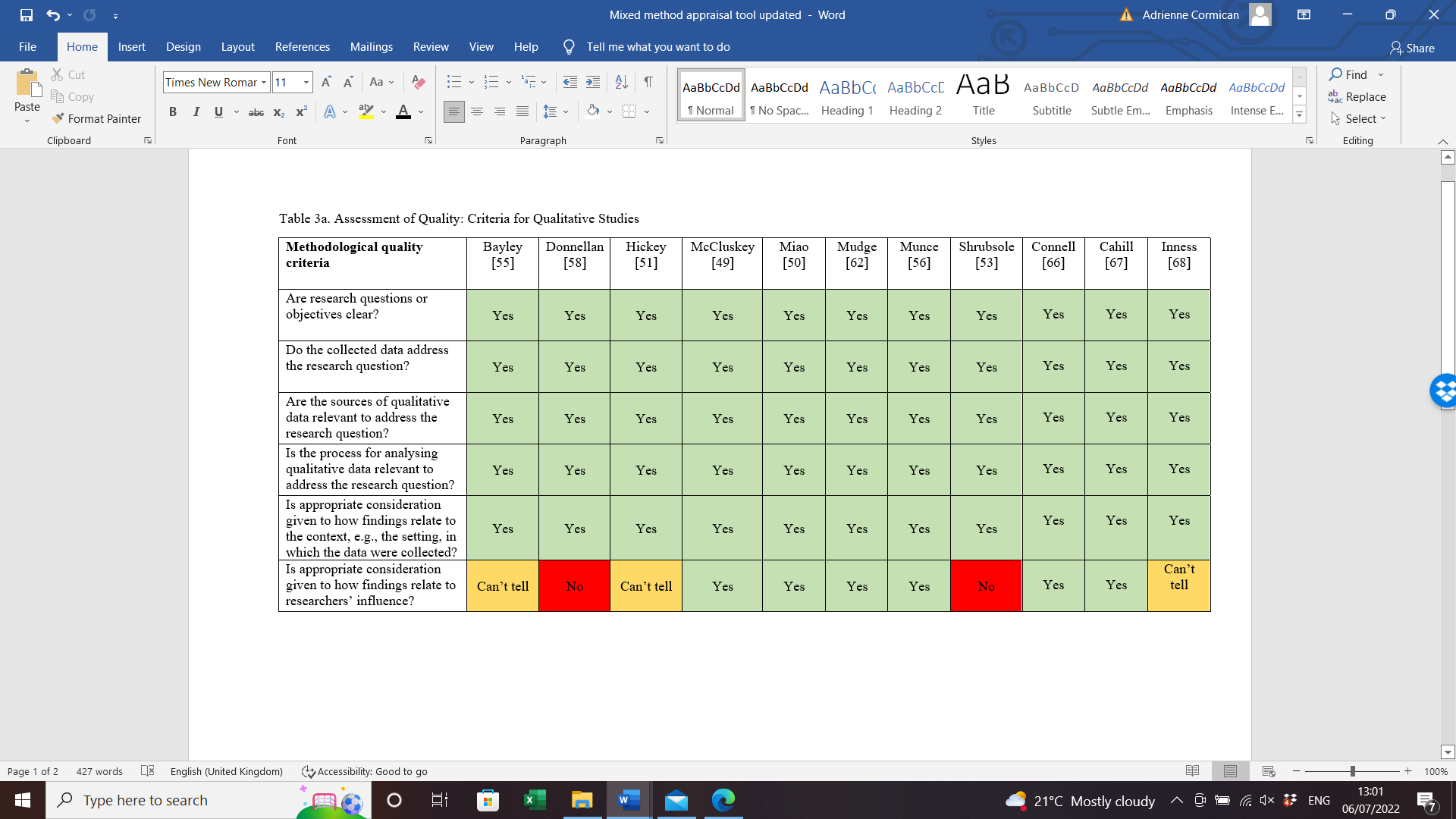


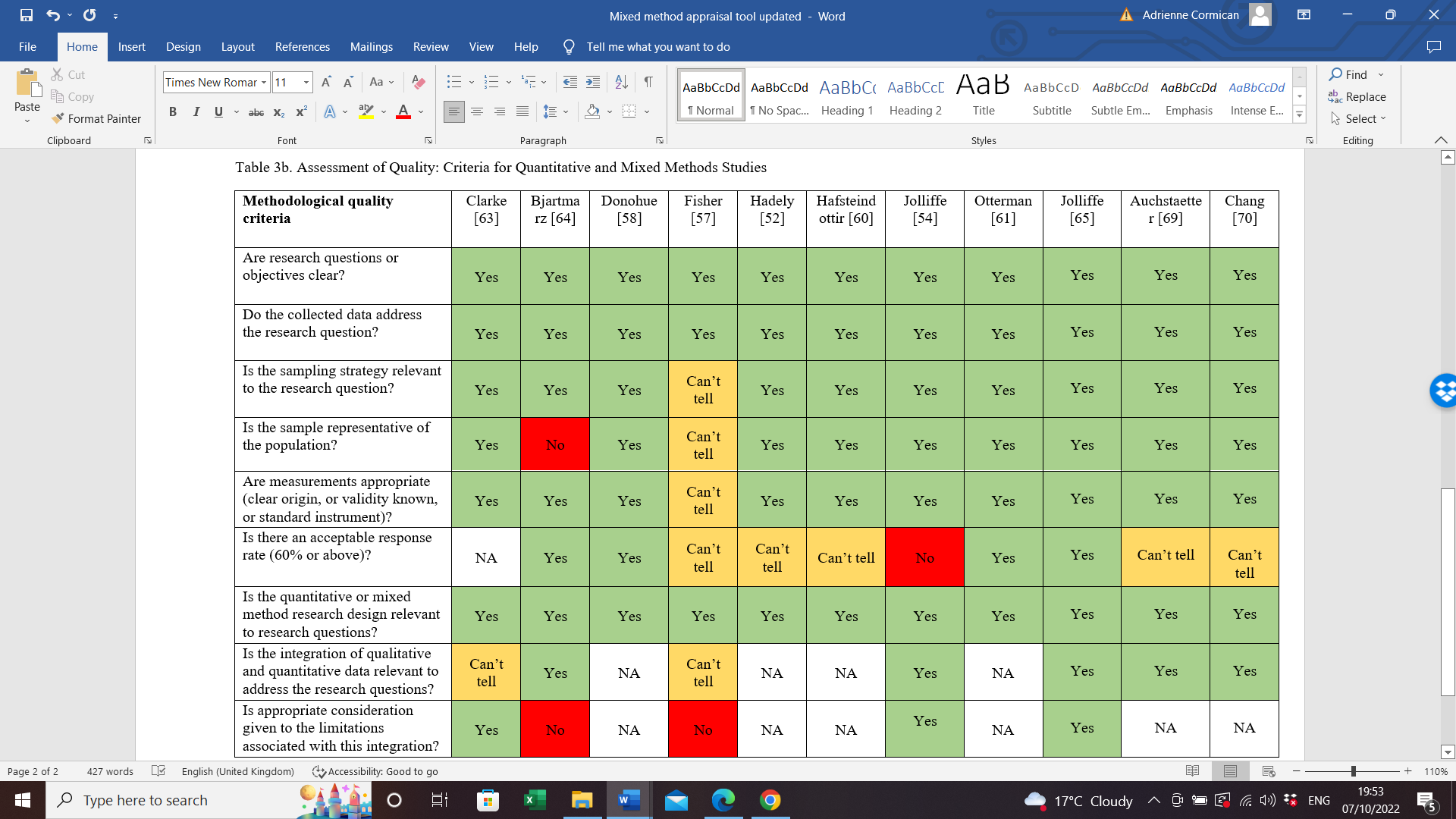

Supplement: sj-docx-1-cre-10.1177_02692155221141036 - Supplemental material for Healthcare professionals’ perceived barriers and facilitators of implementing clinical practice guidelines for stroke rehabilitation: A systematic review [file sj-docx-1-cre-10.1177_02692155221141036.docx]
